# Comprehensive Analysis of an Individualized Immune-Related lncRNA Pair Signature in Gastric Cancer

**DOI:** 10.3389/fcell.2022.805623

**Published:** 2022-02-22

**Authors:** Chuang Nie, Jiabao Zhai, Qi Wang, Xiaojie Zhu, Guanghui Xiang, Chang Liu, Tianyu Liu, Wanyu Wang, Yimin Wang, Yashuang Zhao, Wenjing Tian, Yingwei Xue, Haibo Zhou

**Affiliations:** ^1^ Department of Epidemiology, College of Public Health, Harbin Medical University, Harbin, China; ^2^ Department of Gastroenterological Surgery, Harbin Medical University Cancer Hospital, Harbin, China

**Keywords:** long noncoding RNA, immune, gastric cancer, prognostic signature, immunotherapy

## Abstract

Long noncoding RNAs (lncRNAs) have diverse functions, including immune regulation. Increasing studies have reported immune-related lncRNAs in the prognosis of multiple cancers. In this study, we developed an individualized signature containing 13 immune-related lncRNA pairs (IRLPs) which could predict the overall survival, disease-free survival, progression-free survival, and disease-specific survival of gastric cancer (GC) patients in The Cancer Genome Atlas (TCGA) cohort, and internal and external validations, signature comparisons, and subgroup analyses further confirmed its superiority, stability, and generalizability. Notably, this signature also showed good applicability in discriminating the prognosis of pan-cancer patients. Then, we constructed and validated a nomogram for overall survival based on the signature and clinical factors, which allowed more accurate predictions of GC prognosis. In addition, we revealed that the low survival rate of patients with high-risk scores may be due to their aggressive clinical features, enriched cancer-related signaling pathways, the infiltration of specific immunosuppressive cells, and low tumor mutation burden. We further predicted obviously worse immunotherapeutic responses in the high-risk groups and identified some candidate compounds targeting GC risk group differentiation. This signature based on the IRLPs may be promising for predicting the survival outcomes and immunotherapeutic responses of GC patients in clinical practice.

## Introduction

Gastric cancer (GC) is one of the most prevalent malignancies and the fourth common cause of cancer deaths worldwide with 1.09 and 0.77 million new GC cases and deaths estimated in 2020, respectively(Sung et al., 2021). Due to the insidiousness of the onset of GC, patients are often diagnosed at an advanced stage, with a median overall survival (OS) of less than 12 months when becoming metastatic at distant sites (Digklia and Wagner, 2016). The tumor node metastasis (TNM) staging system is currently the gold standard for guiding the clinical treatment of GC (Egner, 2010). However, patients with the same clinicopathological features and similar therapeutic strategies could exhibit large differences in prognosis because of the complex genetic heterogeneity, which indicates the deficiencies of the TNM staging system in prognostic stratification (Shah and Ajani, 2010). Therefore, novel molecular markers with more clinical utilities are needed to improve prognostic prediction and guide the clinical treatment of GC patients.

Long noncoding RNAs (lncRNAs) are a common type of RNA molecules composed of over 200 nucleotides in length that can regulate gene expression through diverse mechanisms such as chromatin remodeling, genomic imprinting, transcription, and post-transcriptional processing (Rafiee et al., 2018). LncRNAs exert an essential role in the initiation and progression of cancers and have shown great potential as biomarkers in cancer diagnosis, prognosis, and treatment (Zhou et al., 2015; Bhan et al., 2017). More recently, increasing evidence has suggested that lncRNAs participate in tumorigenesis not only by influencing the genome or transcriptome but also by modulating cancer immunity (Denaro et al., 2019). To be specific, lncRNAs could regulate gene expression that is correlated with immune responses, and thus altering immune cell infiltration status in the tumor microenvironment (TME), which could profoundly affect tumor invasiveness, progression, and prognosis (Chen et al., 2017; Lazăr et al., 2018). In recent years, cancer immunotherapy, such as immune checkpoint blockade (ICB), has emerged as a breakthrough for the treatment of many cancers, especially melanoma and non-small-cell lung carcinoma (Postow et al., 2015; Rizvi et al., 2015). Although the clinical trials of ICB in the treatment of patients with advanced GC have achieved some encouraging results, the durable efficacy is limited to a small number of patients, and the objective response rate varies greatly between different studies, which impedes the development of immunotherapies for GC (Smyth et al., 2020; Zeng et al., 2021). Studies have indicated that the immunological components of TME are implicated in the antitumor processes and associated with clinical response to ICB (Lazăr et al., 2018; Zeng et al., 2021). Thus, considering the critical role of lncRNAs in regulating the immune microenvironment, identifying lncRNA signatures that are correlated with immunity may promote our understanding of GC immunobiology and help improve the clinical benefits of immunotherapies.

Using large-scale gene expression data in the public databases, recent studies have developed a variety of immune-related lncRNA based prognostic signatures for various types of cancers, including GC (Shen et al., 2020; Chen et al., 2021; Wang et al., 2021c). However, these studies are not further validated in other independent populations, which may limit their clinical applications in the survival prediction for cancer patients. In addition, these signatures are based on quantifying the expression levels of genes, which need further normalization of model gene expression due to the potential biological heterogeneity among different datasets and inevitable detection biases across different platforms (Leek et al., 2010). Fortunately, two-biomarker combinations based on the relative ranking of gene expression levels could avoid the complexity of data preprocessing and have shown great potential in cancer diagnosis and prognostic prediction (Heinäniemi et al., 2013; Li et al., 2017).

In the present work, we carried out an integrative analysis using public gene expression datasets from various GC cohorts, together with a novel pairing algorithm, to identify and validate an immune-related lncRNA pair signature (IRLPS) for improving prognostic prediction of GC. Then, we explored the underlying biological mechanisms and investigated its correlations with immune cell infiltrations, immunotherapeutic responses, and genomic mutations. In addition, we tried to figure out the potential compounds that might have therapeutic values in GC. Finally, we combined the IRLPS with clinical factors for constructing an individualized nomogram to facilitate clinical applications.

## Materials and Methods

### Data Collection and Preprocessing

The workflow of this study is shown in Supplementary Figure S1. Firstly, we downloaded the uniformly reprocessed RNA-seq data from the *recount2* platform using the “TCGAbiolinks” R package, including 416 GC samples and 37 adjacent normal samples from The Cancer Genome Atlas (TCGA) project and 204 normal gastric samples from The Genotype-Tissue Expression (GTEx) project (Collado-Torres et al., 2017b; Mounir et al., 2019). Then, the raw count gene expression matrix and corresponding transcripts per million (TPM) normalized gene expression matrix was obtained using the “scale_counts” and “getTPM” functions of the “recount” R package, respectively (Collado-Torres et al., 2017a). Genes whose expression level was zero in more than 50% of the samples were filtered out. Clinical information of 381 patients was extracted from the UCSC Xena after excluding those with missing survival or clinical data. TCGA dataset was used as the training cohort. We also downloaded the somatic mutation data of GC patients and gene expression and survival data of TCGA pan-cancer cohort for further analysis. In addition, two external validation GC cohorts, namely the Asian Cancer Research Group (ACRG) cohort (GSE62254, N = 300) and the Singapore cohort (Singapore batches A GSE15459 and B GSE34942, N = 248), were downloaded from the Gene Expression Omnibus (GEO) database. Both of the two external expression datasets were derived from the Affymetrix Human Genome U133 Plus 2.0 (HG-U133 Plus 2.0) array and normalized on the log2 scale. The batch effect in the Singapore cohort was removed using the ComBat algorithm of the “sva” R package as described by Lei et al. (2013) (Leek et al., 2012) All these cohorts have OS and disease-free survival (DFS) data except for the lack of DFS data in the Singapore cohort, while only the TCGA cohort has two other survival indicators, namely progression-free survival (PFS) and disease-specific survival (DSS). The clinical characteristics of each cohort were presented in Supplementary Table S1.

### Immune-Related lncRNA Profile Mining and Differential Expression Analysis

Significant lncRNA-pathway pairs across 33 cancer types were downloaded from the ImmLnc database (Li et al., 2020). Then, the extracted 3,044 lncRNAs related to GC immunity were defined as the immune-related lncRNAs in this study. The lncRNA expression profile in RNA-seq data was extracted according to the GENCODE (release 36) GTF file. For consistency, the microarray lncRNA expression profiles were obtained by re-annotation. Briefly, the probe sets of Affymetrix HG-U133 Plus 2.0 array were aligned to the human genome (GRCh38) with GENCODE v36 annotation using the SeqMap tool with no mismatch (Jiang and Wong, 2008). Then, by keeping the probes uniquely mapped to target gene sequences, a total of 4,231 probes covering 3,346 lncRNAs were obtained. When multiple probes matched with the same lncRNA, the average expression value of these probes was used as the expression level of corresponding lncRNA. To identify differentially expressed immune-related lncRNAs, the “limma” R package was applied to the read count matrix of the 416 GC and 241 normal samples from the *recount2* project, and the thresholds were set as |log2 fold change| (|log2FC|) >1.0 and false discovery rate (FDR) < 0.05 (Ritchie et al., 2015). Since the lncRNA expression profiles in TCGA and GEO databases were different, we focused on the overlapped lncRNAs in the present study.

### Definition and Construction of the Immune-Related lncRNA Pairs

In this study, we used the IRLPs to develop a prognostic signature for GC, in which the expression levels of lncRNAs were pairwise compared in each sample to generate a score for each paired combination. Briefly, if the expression level of lncRNA 1 was higher than that of the lncRNA 2, the score of the IRLP was assigned 1, otherwise, the score was 0. In addition, those IRLPs with a constant value (scores of 0 or 1 assigned to more than 80% of the samples in any dataset) were removed because they may represent the platform-dependent bias measurements and do not contain sufficient discriminative information to predict patients’ survivals.

### Construction and Validation of a Prognostic Signature Based on Immune-Related lncRNA Pairs

Univariate Cox regression analysis was performed in the training cohort based the constructed IRLPs matrix and corresponding clinical data, and prognostic IRLPs with *p* < 0.01 were chosen as the candidates. Then, a Cox regression model that combined with the least absolute shrinkage and selection operator (LASSO) algorithm was further adopted to select a panel of IRLPs to develop the prognostic signature. The optimal tuning parameter was estimated by 10-fold cross-validation at one standard error beyond the minimum partial likelihood deviance. Finally, the selected IRLPs were incorporated into a stepwise based multivariate Cox regression model to construct the IRLPS. The risk score was calculated by adding up each IRLP score, with the weights assigned by the corresponding coefficients generated in the multivariate Cox analysis.

Based on the median risk score in the training cohort, patients in each cohort were classified into the high- and low-risk groups. The Kaplan-Meier method was applied to compare the survival between the two groups. Time-dependent receiver operator characteristic (ROC) curves were adopted to evaluate the predictive performance of the IRLPS. Univariate and multivariate Cox regression analyses were performed to assess the clinical significance of the IRLPS. In addition, stratified analyses were conducted to assess the robustness of the signature in predicting GC prognosis in different clinical subgroups. Furthermore, we compared our IRLPS with four published prognostic signatures that were also constructed using the TCGA data based on both time-dependent ROC and concordance index (C-index) analysis (Wang et al., 2021a; Mao et al., 2021b; Wang et al., 2021c; Xu et al., 2021).

### Functional Annotations and Gene Set Enrichment Analysis

The differentially expressed genes (DEGs) between the high- and low-risk groups were analyzed by the “limma” R package, and the DEGs with |log2FC| >1.0 and FDR<0.05 were subjected to Gene Ontology (GO) and Kyoto Encyclopedia of Genes and Genomes (KEGG) pathway enrichment analyses by using the “clusterProfiler” R package (Yu et al., 2012). In addition, we performed a pre-ranked Gene Set Enrichment Analysis (GSEA) to identify the cancer hallmarks and immunologic signatures that are correlated with the IRLPS (Subramanian et al., 2005). The hallmark gene sets (h.all.v7.4. entrez.gmt) and immunologic gene sets (c7. all.v7.4. entrez.gmt) were obtained from the Molecular Signatures Database. Gene sets with nominal *p* < 0.05 and FDR < 0.25 were considered significantly enriched.

### Exploration of Immunological Features

Several generally acknowledged methods, including CIBERSORT, EPIC, MCP-counter, xCell, ESTIMATE were applied to analyze the infiltrating characteristics of immune cells and stromal cells based on the gene expression data of tumor tissues (Yoshihara et al., 2013; Newman et al., 2015; Becht et al., 2016; Aran et al., 2017; Racle et al., 2017). Then, the correlations between the IRLPS and immune/stromal cells were calculated by Spearman correlation analysis. In addition, since immune checkpoint gene expression may be correlated with the treatment responses of ICB therapy, we further assessed the associations between the IRLPS and several key immune checkpoint molecules (Goodman et al., 2017).

### Prediction of Immunotherapeutic Response

To explore the response of immunotherapy in different risk groups, we applied the Tumor Immune Dysfunction and Exclusion (TIDE) algorithm to generate predicted results about ICB therapy (anti-PD1 and anti-CTLA4) for each GC patient (Jiang et al., 2018; Fu et al., 2020). Given that tumor mutation burden (TMB) has emerged as a promising biomarker for predicting the immunotherapeutic response, we also calculated the TMB of GC patients in the TCGA cohort. TMB was defined as the total number of nonsynonymous mutations per megabase in the coding area of a tumor genome (Wang et al., 2021b). The “maftools” and “GenVisR” R packages were used to analyze and visualize common mutations (Skidmore et al., 2016; Mayakonda et al., 2018).

### Calculation of Stemness Index

Cancer stemness has been reported to be associated with suppressed immune response and significantly poor outcome for most cancers (Miranda et al., 2019). Thus, we investigated whether the established IRLPS was correlated with the stemness phenotype of GC. As previously reported, we utilized the one-class logistic regression (OCLR) algorithm to calculate the RNA expression-based stemness index (mRNAsi) for each tumor sample (Malta et al., 2018). The mRNAsi was mapped to the range of 0–1 through a linear transformation that subtracted the minimum and divided by the maximum.

### Connectivity Map Analysis

To explore potential compounds that might be useful for GC treatment, the top 1,000 DEGs (FDR<0.05) between high- and low-risk groups were employed to query the Connectivity Map (CMap) database (Subramanian et al., 2017). Then, compounds with a negative enrichment score and *p* < 0.05 were selected for the subsequent mechanism of action (MoA) analysis.

### Construction and Validation of an Individualized Nomogram

To facilitate the clinical utilization of the IRLPS, we established a nomogram in the TCGA cohort by integrating the IRLPS with independent clinical prognostic factors from multivariate Cox regression. Then, the nomogram was applied to the ACRG cohort for external validation (the Singapore cohort was not included due to the lack of corresponding clinical data). C-index and time-dependent ROC analyses were both applied to evaluate the performance of the nomogram. Moreover, calibration curves were used to display the differences between the actual observed and predicted survival possibility. Decision curve analyses (DCA) were used to assess the net benefits of different clinical decisions.

### Statistical Analysis

All the statistical analyses were conducted on the R software (version 4.0.0). The R packages used in this study are listed in Supplementary Table S2. Internal validation was performed using the bootstrap method. The Wilcoxon rank-sum test and Kruskal-Wallis test were used to evaluate the correlations of IRLPS score with clinical factors, as well as the correlations of other evaluated factors (TIDE score, mRNAsi, and TMB et al.) with IRLPS groups. The chi-square test was used to analyze the differences of specific gene mutations among IRLPS groups. In addition, Spearman correlation analyses were performed to investigate the relationship between IRLPS score and immune cells and immune checkpoints. *p* < 0.05 was considered statistically significant. Bonferroni correction was performed in the subgroup analysis.

## Results

### Differentially Expressed Immune-Related lncRNAs

A total of 970 differentially expressed immune-related lncRNAs were identified in GC, among which 429 were upregulated and 541 were downregulated (Supplementary Figure S2A). Then, we crossed these lncRNAs with 3,346 re-identified lncRNAs in the microarray datasets to obtain 312 shared lncRNAs for the following analysis (Supplementary Figure S1 and Supplementary Figure S2B), and the details were shown in Supplementary Table S3.

### Construction and Internal Evaluation of the Immune-Related lncRNA Pairs

**FIGURE 1:**
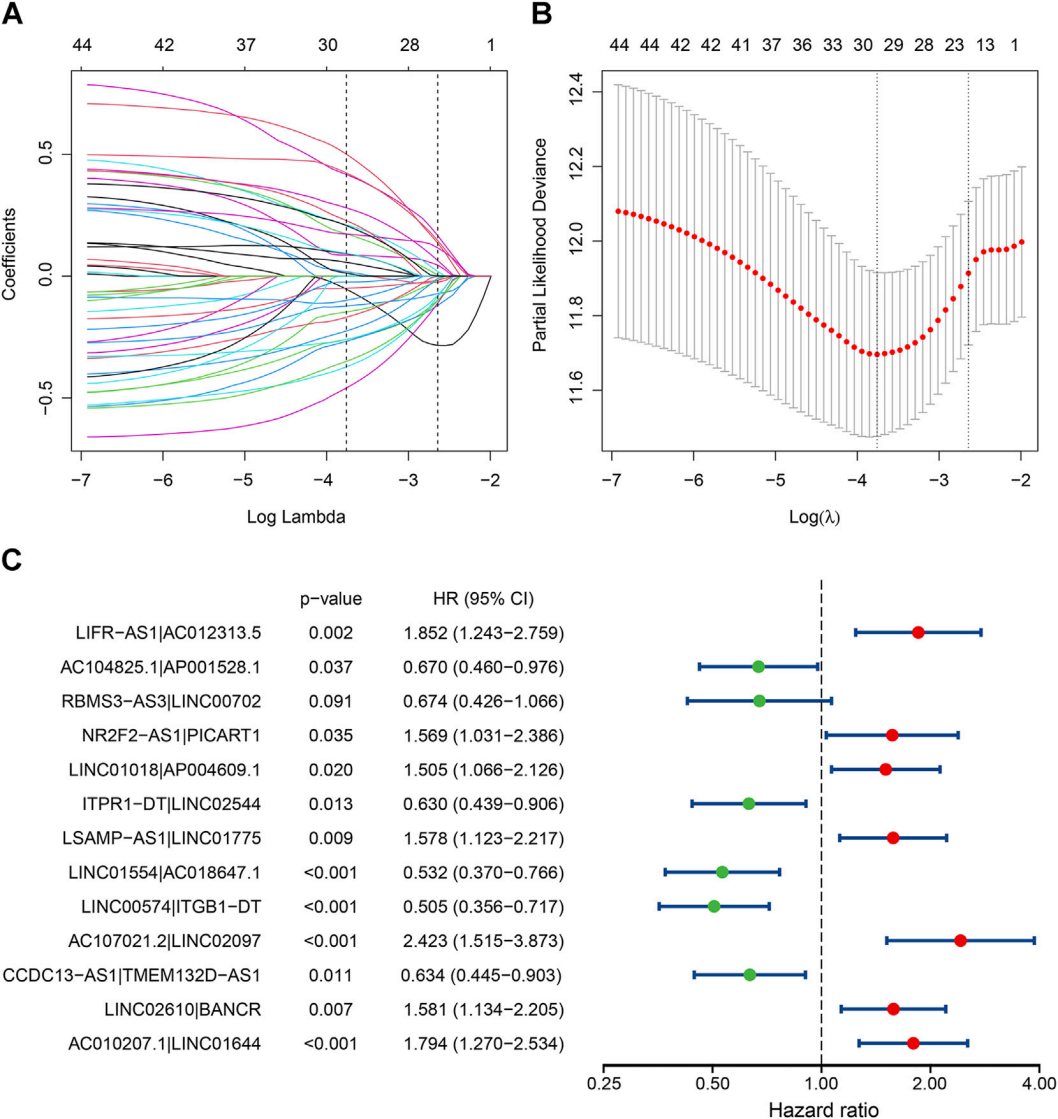
Identification of the prognostic signature in the TCGA cohort. **(A)** The least absolute shrinkage and selection operator (LASSO) coefficient profiles of the prognostic immune-related lncRNA pairs (IRLPs); **(B)** The 10-fold cross-validated partial likelihood deviance. The lambda value was confirmed as 0.07124 at one standard deviation of the minimal deviance, which resulted in 20 non-zero coefficients; **(C)** The forest map showed 13 IRLPs identified by multivariate Cox proportional hazard regression in the stepwise method.

A total of 48,516 IRLPs were built by pairwise comparison using the 312 shared lncRNAs, and 2,186 of them were kept after excluding those with a constant value. With univariate Cox analysis, 44 OS-related IRLPs were identified in the training cohort (*p* < 0.01) (Supplementary Table S4). Then, these prognostic IRLPs were subjected to LASSO-Cox regression analysis. The LASSO coefficient profiles and partial likelihood deviance plot were shown in Figures 1A,B, illustrating the selected 20 IRLPs. Then, we further applied the multivariate Cox analysis and ultimately obtained 13 IRLPs for constructing the IRLPS and generating the risk scores (Figure 1C).

**FIGURE 2:**
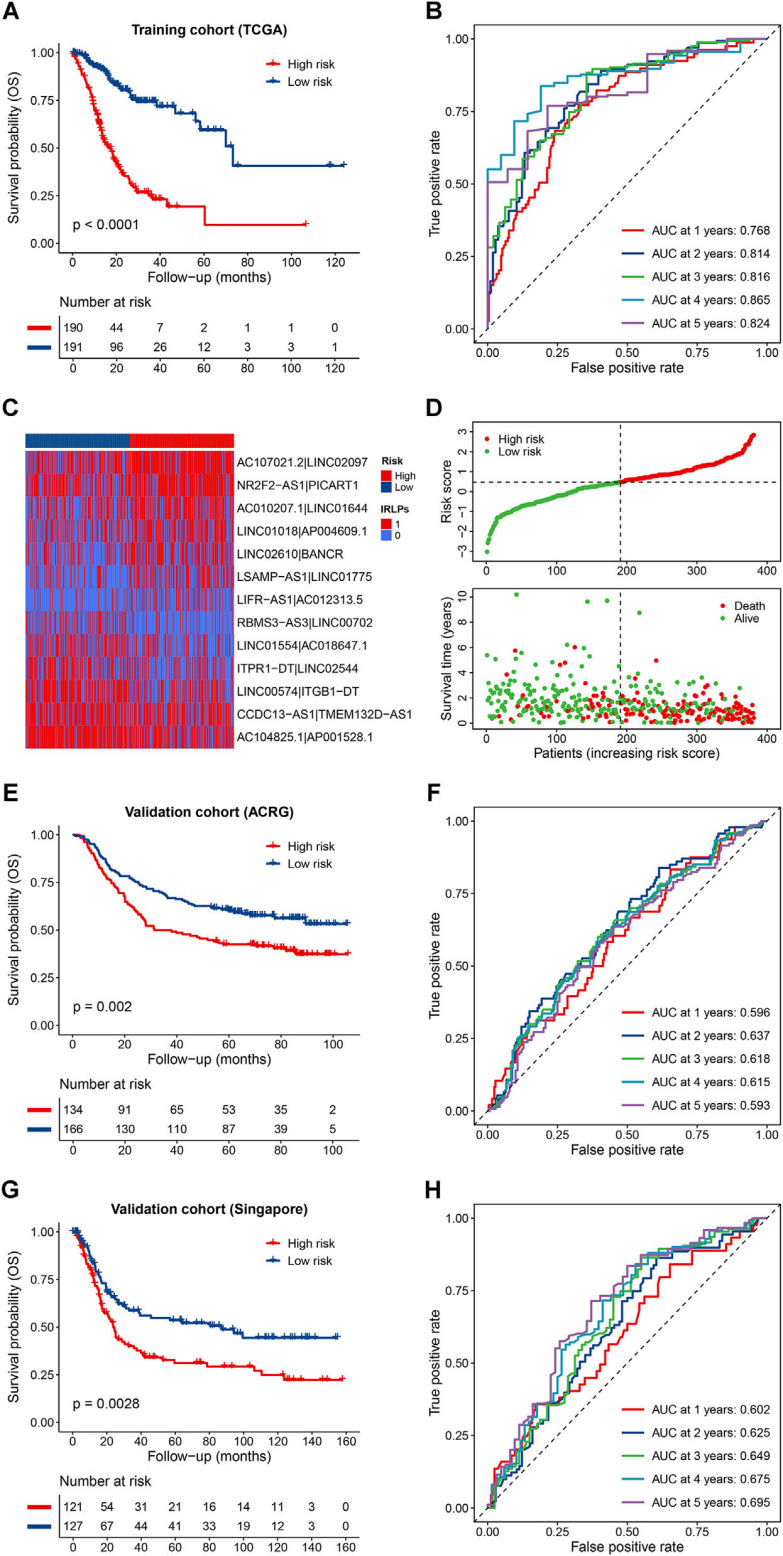
Predictive effects of the prognostic signature. **(A,B)** Kaplan-Meier curves and time-dependent receiver operator characteristic (ROC) curves of the signature for predicting the overall survival (OS) of patients in the TCGA cohort; **(C,D)** Heatmap of the immune-related lncRNA pairs (IRLPs) in the high- and low-risk groups, rank of the risk score, and distribution of the survival status; **(E,F)** Kaplan-Meier curves and time-dependent ROC curves of the signature for predicting the OS of patients in the Asian Cancer Research Group (ACRG) cohort; **(G,H)** Kaplan-Meier curves and time-dependent ROC curves of the signature for predicting the OS of patients in the Singapore cohort.

Based on the median cutoff value, patients in the training cohort were divided into the high- and low-risk groups. The Kaplan-Meier survival curves revealed that patients in the high-risk group had significantly poorer OS than those in the low-risk group (*p* < 0.0001) (Figure 2A). Time-dependent ROC analysis suggested that our IRLPS had a good performance in predicting OS of GC patients with a 5-years average area under the curve (AUC) of 0.817 (Figure 2B). The heatmap of the 13 IRLPs showed that a score of 1 in AC107021.2|LINC02097, NR2F2-AS1|PICART1, AC010207.1|LINC01644, LINC01018|AP004609.1, LINC02610|BANCR, LSAMP-AS1|LINC01775, and LIFR-AS1|AC012313.5 was more distributed in the high-risk group, indicating their harmful roles in GC prognosis, while the remaining IRLPs showed the reverse trend (Figure 2C). In addition, the distribution of the risk scores also indicated poor survival in the high-risk group patients (Figure 2D). Interestingly, we further found that patients in the high-risk groups had a poorer DFS, PFS, and DSS compared with those in the low-risk groups (all *p* < 0.0001), and the high predictive ability of the IRLPS was revealed by the time-dependent ROC analysis (5-years average AUCs of 0.737, 0.764 and 0.813, respectively) (Supplementary Figures S3A–S3F). Moreover, internal validations by bootstrapping verified the performance of the signature in predicting different survival indicators (Supplementary Table S5).

### External Validation of the Immune-Related lncRNA Pairs

To assess the robustness of the IRLPS in the independent external population, the formula and the cutoff value used in the training cohort were applied to two GEO datasets. The results showed that patients in the high-risk groups also exhibited obviously worse OS than those in the low-risk groups for both the ACRG (*p* = 0.002) and Singapore cohort (*p* = 0.0028) (Figures 2E,G), and the corresponding 5-years average AUC values were 0.612 and 0.649, respectively (Figures 2F,H). Similar results were observed in the entire GEO validation cohort and the whole cohort (all *p* < 0.0001; 5-years average AUCs of 0.625 and 0.679, respectively) (Supplementary Figures S4A–S4D). In terms of the DFS, the discriminative ability of the IRLPS was also validated in the ACRG cohort (*p* = 0.0031; 5-years average AUC of 0.613) and the combined cohort (*p* < 0.0001; 5-years average AUC of 0.644) (Supplementary Figures S5A–S5D). Notably, we were pleasantly surprised to find that the IRLPS could also predict all survival indicators of digestive tract cancers and even pan-cancer with corresponding low-risk groups showed survival advantages (all *p* < 0.01) (Supplementary Figures S6A–S6H). Furthermore, compared with other traditional prognostic signatures, the IRLPS showed an obviously higher accuracy for OS prediction in GC patients as demonstrated by the ROC and C-index analyses (Supplementary Figures S7A, S7B).

### Subgroup Analyses

According to the clinical traits, including age, gender, tumor stage, grade, T stage, N stage, M stage, and Lauren subtype, GC patients in both separate and combined cohorts were divided into sixteen subgroups. As shown in Supplementary Table S6, the IRLPS could significantly discriminate the OS of patients in every subgroup of the TCGA cohort (all *p* < 0.025), and similar results were observed in the validation cohorts especially for the male, advanced stage (stage III-IV), N1-3 stage, M0 stage, and intestinal adenocarcinoma groups (all *p* < 0.025). In addition, we further performed subgroup analyses in the DFS datasets and suggested that the IRLPS remained an indicator of the DFS in the younger (<60 years), male, advanced stage, high grade (G3), N1-3 stage, M0 stage, and intestinal adenocarcinoma groups (all *p* < 0.025) (Supplementary Table S7).

### Independent Prognostic Role of the Immune-Related lncRNA Pairs

To investigate whether the IRLPS was an independent prognostic factor in GC, we conducted the Cox regression analysis. Univariate Cox regression analysis indicated that the high-risk groups had poorer OS than the low-risk groups in the TCGA (HR = 4.541, 95% CI: 3.129–6.591, *p* = 1.68 × 10^−15^), ACRG (HR = 1.646, 95% CI: 1.196–2.264, *p* = 0.002), and Singapore cohorts (HR = 1.726, 95% CI: 1.201–2.479, *p* = 0.003) (Table 1). After adjusting for the clinical features in the multivariate Cox analysis, we found that the IRLPS was an independent prognostic factor in the TCGA (HR = 4.549, 95% CI: 3.096–6.685, *p* = 1.22 × 10^−14^), ACRG (HR = 1.646, 95% CI: 1.190–2.277, *p* = 0.003), and Singapore cohorts (HR = 1.578, 95% CI: 1.093–2.278, *p* = 0.015) (Table 1). In addition, the IRLPS was also independently associated with the DFS of GC patients in the TCGA (HR = 3.342, 95% CI: 1.804–6.192, *p* = 1.26 × 10^−4^) and ACRG cohorts (HR = 1.600, 95% CI: 1.118–2.290, *p* = 0.010) (Supplementary Table S8).

**TABLE 1:** Univariate and multivariate Cox regression analysis of clinicopathologic factors and IRLPS for predicting OS in the training and validation cohorts.

For OS variables	Univariate analysis		Multivariate analysis	
	HR (95% CI)	*p* Value	HR (95% CI)	*p* Value
TCGA training cohort (N = 381)^a^				
Age (≥60 vs. <60 years)	1.574 (1.084–2.286)	0.017	1.740 (1.186–2.553)	0.005
Gender (Male vs. Female)	1.216 (0.861–1.718)	0.267	—	—
Histological grade (G3 vs. G1-2)	1.436 (1.016–2.030)	0.040	1.345 (0.945–1.916)	0.100
T stage (T3-4 vs. T1-2)	1.748 (1.157–2.643)	0.008	1.362 (0.857–2.163)	0.191
N stage (N1-3 vs. N0)	2.014 (1.342–3.022)	7.21e-04	1.017 (0.609–1.700)	0.948
M stage (M1 vs. M0)	2.213 (1.272–3.849)	0.005	2.242 (1.254–4.007)	0.006
Tumor stage (Stage III-IV vs. Stage I-II)	2.057 (1.456–2.905)	4.25e-05	1.652 (1.027–2.657)	0.039
IRLPS (high- vs. low-risk)	4.541 (3.129–6.591)	1.68e-15	4.549 (3.096–6.685)	1.22e-14
ACRG validation cohort (N = 300)				
Age (≥60 vs. <60 years)	1.239 (0.882–1.741)	0.217	—	—
Gender (Male vs. Female)	0.905 (0.647–1.265)	0.559	—	—
T stage (T3-4 vs. T1-2)	2.396 (1.741–3.297)	8.22e-08	1.336 (0.902–1.981)	0.149
N stage (N1-3 vs. N0)	2.816 (1.434–5.527)	0.003	1.729 (0.847–3.531)	0.133
M stage (M1 vs. M0)	3.840 (2.482–5.942)	1.52e-09	2.989 (1.872–4.772)	4.51e-06
Lauren subtype (Diffused vs. Intestinal)	1.677 (1.198–2.347)	0.003	1.130 (0.789–1.617)	0.505
Lauren subtype (Mixed vs. Intestinal)	2.128 (1.167–3.879)	0.014	1.705 (0.928–3.131)	0.086
Tumor stage (Stage III-IV vs. Stage I-II)	3.408 (2.341–4.960)	1.53e-10	2.092 (1.293–3.386)	0.003
IRLPS (high- vs. low-risk)	1.646 (1.196–2.264)	0.002	1.646 (1.190–2.277)	0.003
Singapore validation cohort (N = 248)				
Age (≥60 vs. <60 years)	0.973 (0.659–1.437)	0.889	—	—
Gender (Male vs. Female)	1.128 (0.775–1.639)	0.530	—	—
Lauren subtype (Diffused vs. Intestinal)	1.308 (0.896–1.911)	0.164	—	—
Lauren subtype (Mixed vs. Intestinal)	0.948 (0.471–1.907)	0.880	—	—
Tumor stage (Stage III-IV vs. Stage I-II)	6.136 (3.725–10.110)	1.05e-12	5.995 (3.633–9.892)	2.40e-12
IRLPS (high- vs. low-risk)	1.726 (1.201–2.479)	0.003	1.578 (1.093–2.278)	0.015

IRLPS: immune-related lncRNA, pair signature; OS: overall survival; HR: hazard ratio; CI: confidence interval; TCGA: The cancer genome atlas, ACRG: Asian cancer research group.

a Patients with Gx (histological grade cannot be assessed) or Nx (lymph nodes cannot be assessed) or Mx (distant metastasis cannot be assessed) were included in the analysis but were not displayed in this table.

### Clinical Correlation Analysis for the Immune-Related lncRNA Pairs

**FIGURE 3:**
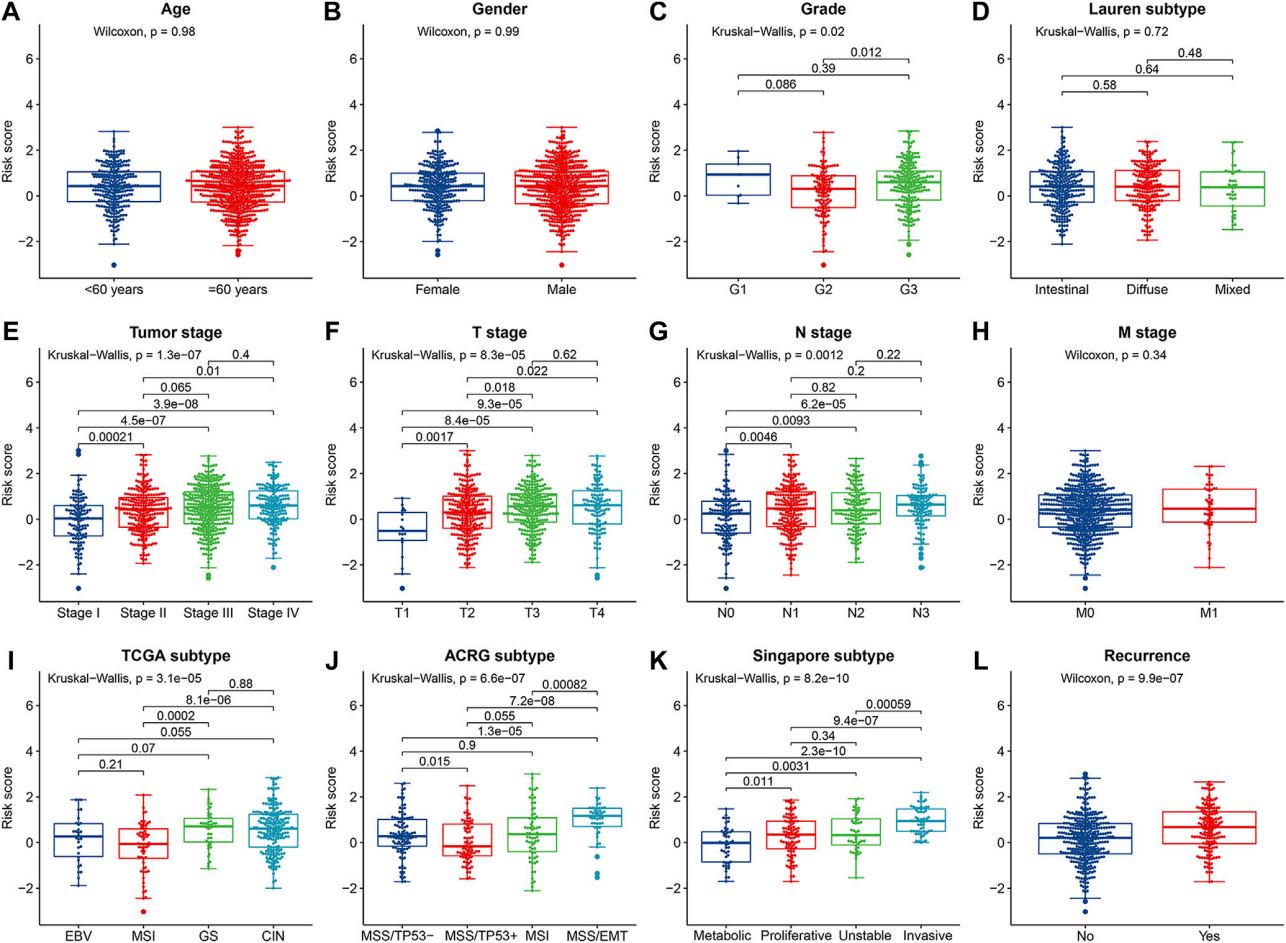
The correlations between the prognostic risk score and **(A)** age; **(B)** gender; **(C)** tumor grade; **(D)** Lauren subtype; **(E)** tumor stage; **(F)** T stage; **(G)** N stage; **(H)** M stage; **(I)** TCGA subtype; **(J)** Asian Cancer Research Group (ACRG) subtype; **(K)** Singapore subtype; and **(L)** recurrence status.

The correlations between the IRLPS and the clinicopathological features were analyzed in the whole cohort. The results showed that the IRLPS was not associated with age, gender, M stage, and Lauren subtype. However, we found significant correlations between the IRLPS and tumor grade (*p* = 0.020), tumor stage (*p* = 1.30 × 10^−7^), T stage (*p* = 8.30 × 10^−5^), N stage (*p* = 0.0012), recurrence status (*p* = 9.90 × 10^−7^), and the molecular subtypes of TCGA (*p* = 3.10 × 10^−5^), ACRG (*p* = 6.60 × 10^−7^), and Singapore cohorts (*p* = 8.20 × 10^−10^) (Figures 3A–3L), suggesting that the IRLPS may be involved in the progression of GC.

### Functional Enrichment Analysis

**FIGURE 4:**
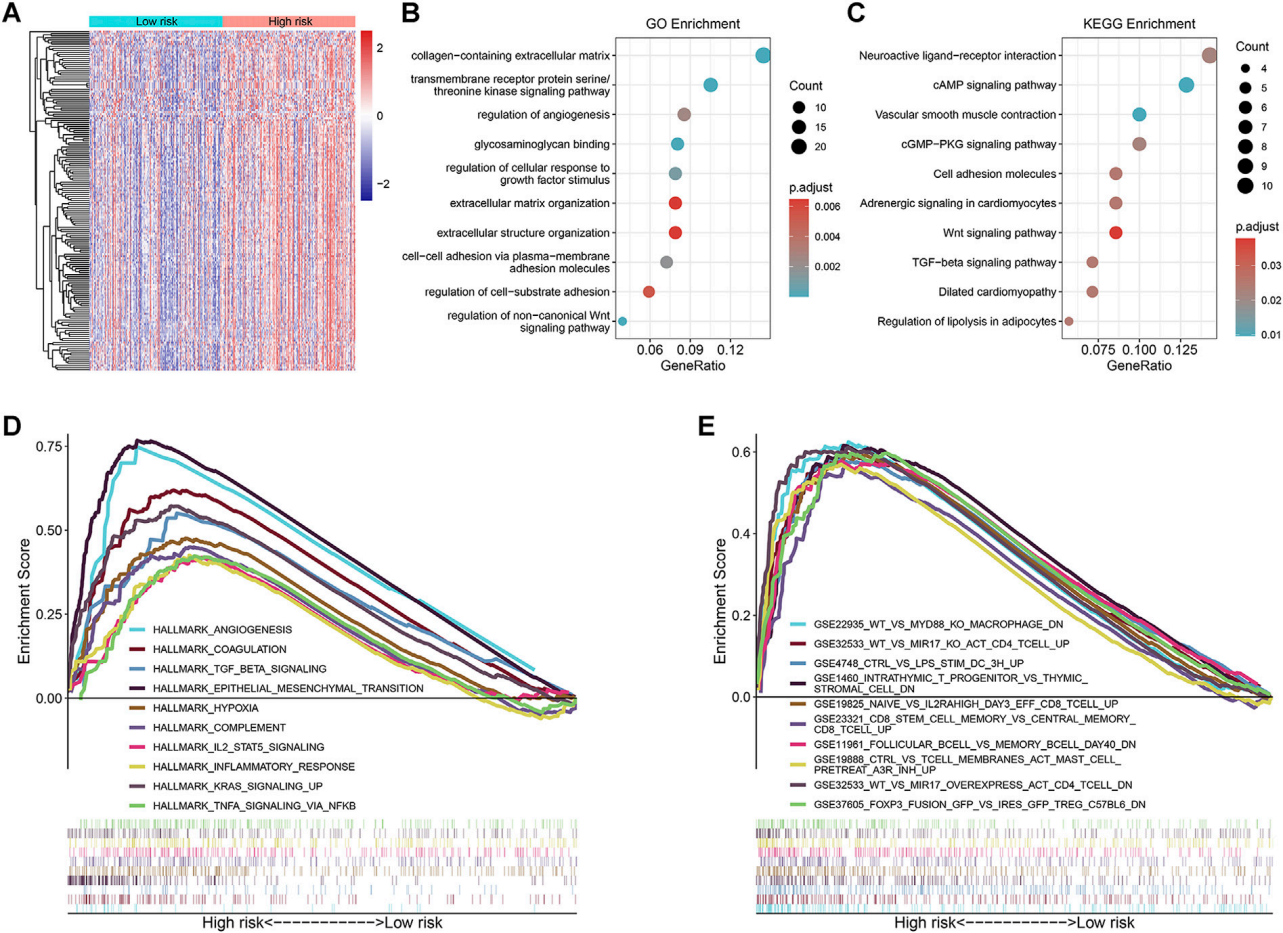
Exploration of the underlying molecular mechanisms of the prognostic signature. **(A)** Heatmap of the differentially expressed genes (DEGs) between the high- and low-risk groups; **(B,C)** Significantly enriched Gene Ontology (GO) annotations and Kyoto Encyclopedia of Genes and Genomes (KEGG) pathways by analyzing the DEGs up-regulated in the high-risk group; **(D,E)** Pre-ranked gene set enrichment analysis (GSEA) of the tumor hallmarks and immunologic characteristics correlated with the prognostic signature.

Using gene expression data of the TCGA cohort, 165 significant DEGs were identified between the high- and low-risk groups (Figure 4A). Among them, 163 genes were up-regulated in the high-risk group and only 2 genes were down-regulated (Supplementary Table S9). Therefore, we conducted GO annotation and KEGG enrichment analysis using the up-regulated DEGs and found that these genes were enriched in GO terms such as collagen-containing extracellular matrix (ECM), regulation of angiogenesis, and regulation of cellular response to growth factor stimulus (Figure 4B and Supplementary Table S10), and KEGG pathways such as cAMP, cGMP-PKG, and TGF-β signaling (Figure 4C and Supplementary Table S11). In addition, subsequent pre-ranked GSEA of tumor hallmarks revealed that the high-risk group also exhibited an obvious enrichment of common pathways including angiogenesis, epithelial-mesenchymal transition (EMT), and TGF-β signaling (Figure 4D and Supplementary Table S12). Moreover, we observed the enrichment of numerous immunologic signatures such as follicular B cells vs. memory B cells down and naive vs. effector CD8 T cells up in the high-risk group (Figure 4E and Supplementary Table S13), which implied the immune-related modulation of the signature.

### Estimation of the Immune Cell Infiltration and Immunosuppressive Molecules With Immune-Related lncRNA Pairs

**FIGURE 5:**
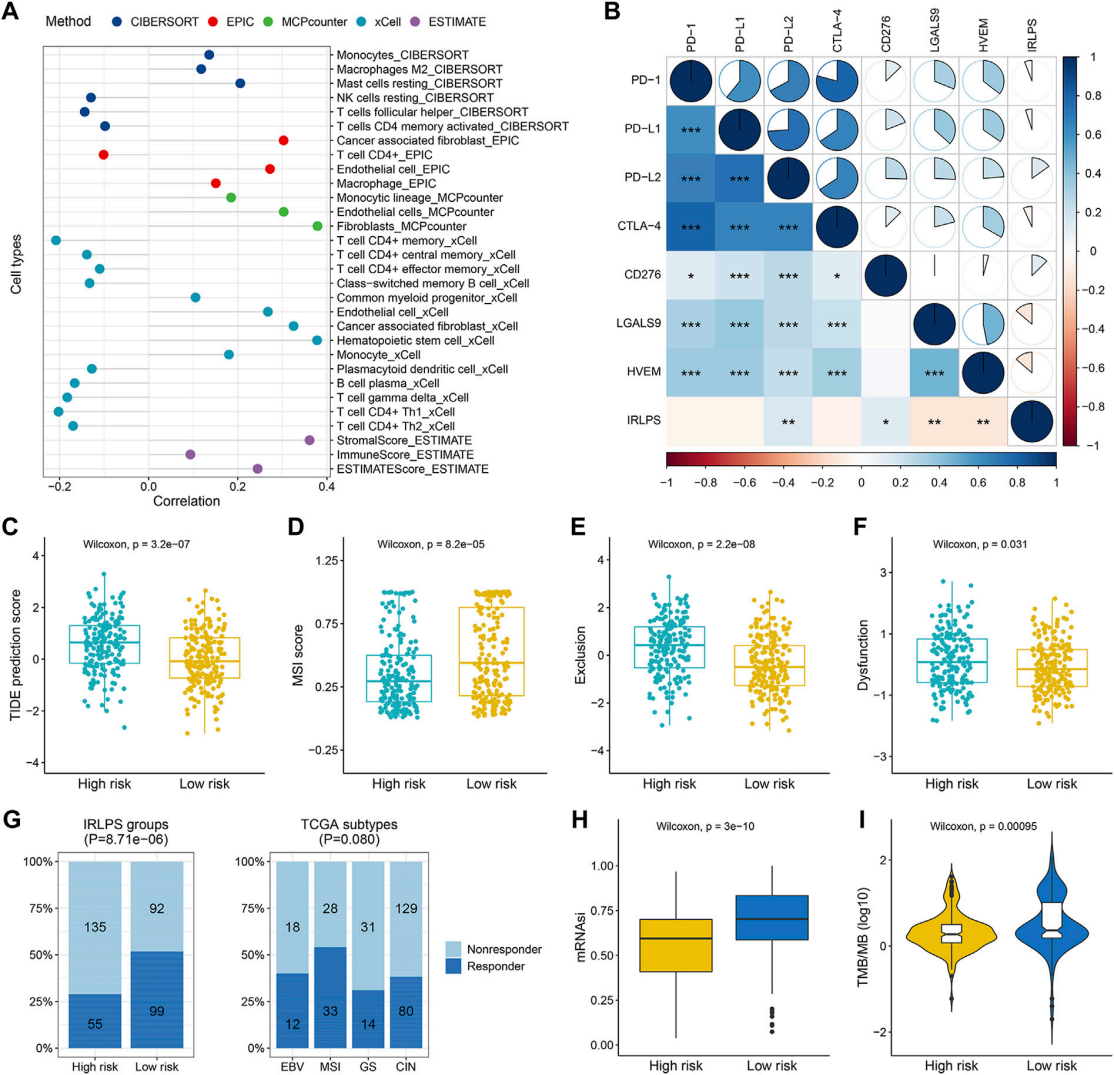
The correlations between the tumor-infiltrating immune cells, immunosuppressed molecules, predicted immunotherapeutic responses, and our prognostic signature in the TCGA cohort. **(A)** Lollipop plot displayed the correlations between the signature and tumor-infiltrating immune cells estimated by different algorithms; **(B)** Correlogram showed the correlations between the signature and several crucial immune checkpoint genes, including *PD-1*, *PD-L1*, *PD-L2*, *CTLA-4*, *CD276*, *LGALS9*, and *HVEM* (correlation coefficients is represented by the area and colored according to the value; *p < 0.05, **p < 0.01, ***p < 0.001); **(C–F)** Comparisons of the Tumor Immune Dysfunction and Exclusion (TIDE) scores, T cell exclusion scores, dysfunction scores, and microsatellite instability (MSI) scores between the high- and low-risk groups; **(G)** Comparisons of the proportions of predicted responders and non-responders to immunotherapy among different risk groups (left panel) and TCGA subtypes (right panel); **(H)** Boxplot demonstrated the higher stemness index (mRNAsi) in the low-risk group; **(I)** Violin plot showed the significant difference in the tumor mutation burden (TMB) between the high- and low-risk groups.

Since the IRLPS were initially connected with immune pathways, we consequently explored whether this signature was correlated with tumor immune microenvironment based on the gene expression data of the three cohorts. We found that activated tumor-infiltrating immune cells such as CD4^+^ T cells and T helper cells were more negatively correlated with this signature, while monocytes, macrophages, mast cells, endothelial cells, and cancer-associated fibroblasts showed positive correlations in the TCGA cohort (all *p* < 0.05) (Figure 5A). Similar results were observed in the ACRG and Singapore cohorts (Supplementary Figure S8A and Suppplementary Figure S9A). In addition, the stromal score from the ESTIMATE algorithm was positively associated with the IRLPS, while the immune score showed no correlation (Figure 5A, Supplementary Figure S8A and Suppplementary Figure S9A). The above results are detailed in Supplementary Table S14. Furthermore, we found that the IRLPS score was generally negatively correlated with the expression of *CLTA-4*, *LGALS9*, and *HVEM* (all *p* < 0.05) and positively correlated with *PD-L2* and *CD276* in the three cohorts (all *p* < 0.05), but no significant associations were observed between *PD-1* and *PD-L1* expression and the signature (Figure 5B, Supplementary Figure S8B and Suppplementary Figure S9B).

### Prediction of the Response to Immunotherapy in Different Risk Groups

Using the TIDE algorithm, we found that the high-risk group had a higher TIDE score than the low-risk group (*p* = 3.2 × 10^−7^) (Figure 5C), suggesting that the high-risk patients were less likely to benefit from ICB therapy, which was in line with the lower predicted proportion of responders in the high-risk group (28.95 vs. 51.83%, *p* = 8.71 × 10^−6^) (Figure 5G). In addition, we found that the high-risk group had higher T cell exclusion and dysfunction scores but lower microsatellite instability scores (all *p* < 0.05) (Figures 5D–F). Consistent prediction results were observed in both the ACRG and Singapore cohorts regarding the immunotherapy response rates, TIDE scores, and T cell exclusion scores (all *p* < 0.05) (Supplementary Figures S8C–S8G and Supplementary Figures S9C–S9G). Besides, we observed obvious differences in the distribution of predicted responders across the ACRG (*p* = 2.32 × 10^−8^) and Singapore molecular subtypes (*p* = 7.75 × 10^−8^), with the EMT (2.17%) and invasive subtypes (8.33%) exhibited the worst response rates, respectively (Supplementary Figure S8G and Suppplementary Figure S9G). No significant difference was found regarding the TCGA subtypes (*p* = 0.080) (Figure 5G). Interestingly, we observed higher mRNAsi in the low-risk-groups compared with the high-risk groups (all *p* < 0.01) (Figure 5H, Supplementary Figure S8H and Suppplementary Figure S9H), implying the better prognosis of patients with higher mRNAsi as previously reported (Mao et al., 2021a). Furthermore, patients in the low-risk group had obviously higher TMB than those in the high-risk group (*p* = 0.00095) (Figure 5I), which further supported that the low-risk patients may have enhanced responses in ICB therapy.

### Somatic Mutation Analysis

**FIGURE 6:**
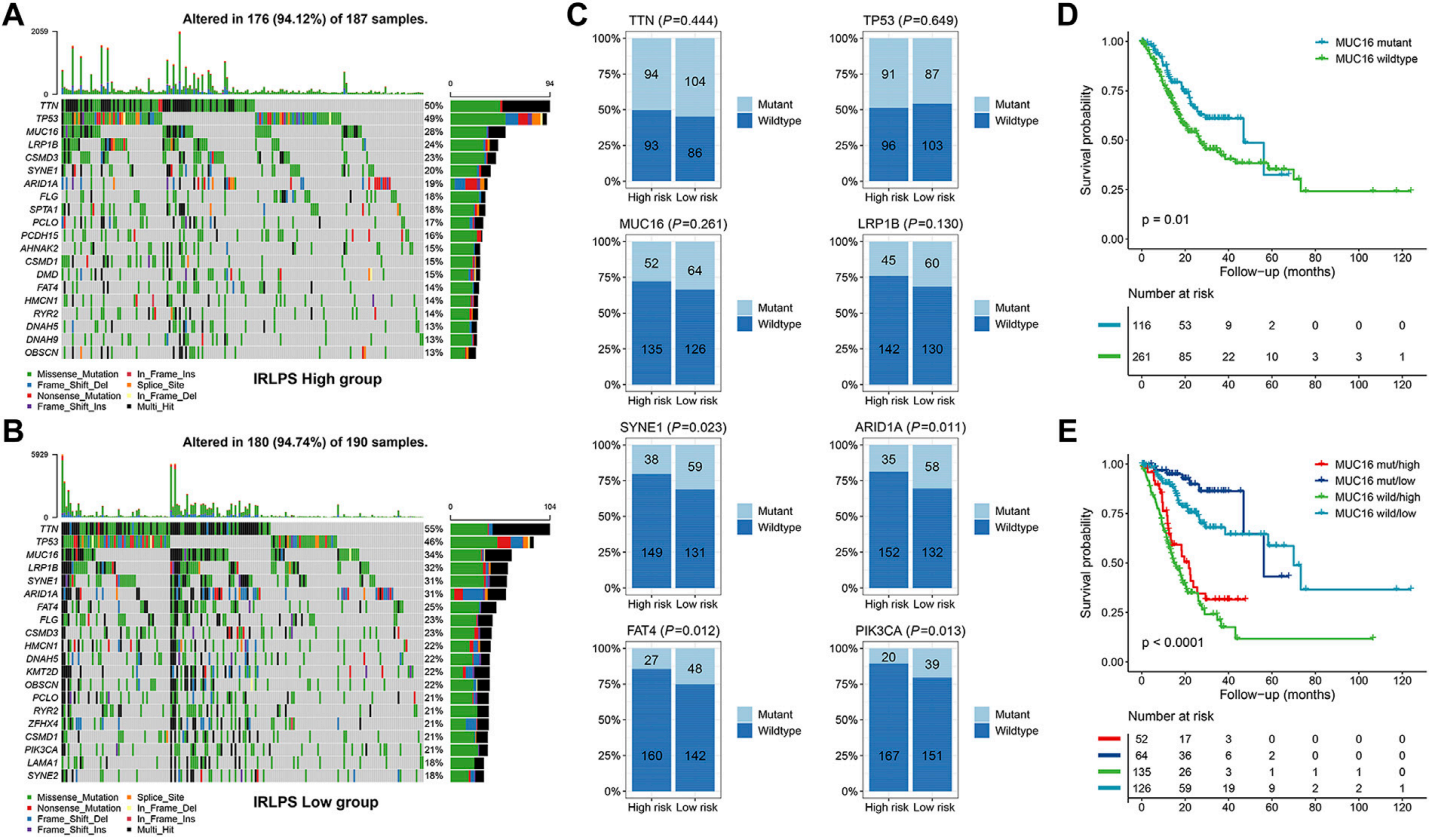
Comparisons of the somatic mutations between the high- and low-risk groups of the TCGA cohort. **(A,B)** Waterfall plot displayed the top 20 mutated genes in the high- and low-risk groups, respectively; **(C)** Comparisons of the mutation status of *TTN*, *TP53*, *MUC16*, *LRP1B*, *SYNE1*, *ARID1A*, *FAT4*, and *PIK3CA* between the high- and low-risk groups; **(D)** Kaplan-Meier curve analysis of the overall survival (OS) stratified by the *MUC16* mutation status; **(E)** Kaplan-Meier curve analysis of the OS according to both the *MUC16* mutation status and our prognostic signature.

Next, we performed somatic mutation analysis to investigate the genomic differences between the two risk groups of the TCGA cohort. The waterfall plot presented the top 20 genes with the highest mutation frequency in the high- and low-risk groups (Figures 6A,B). Therein, *TTN*, *TP53*, *MUC16*, and *LRP1B* were the most mutated genes in both the groups but no significant differences were found for their mutation frequencies (all *p* > 0.05) (Figure 6C). However, other common mutated genes in GC such as *SYNE1* (*p* = 0.023), *ARID1A* (*p* = 0.011), *FAT4* (*p* = 0.012), and *PIK3CA* (*p* = 0.013) exhibited significantly higher mutation frequencies in the low-risk patients (Figure 6C). *MUC16* mutations were correlated with favorable survival outcomes in GC patients (Figure 6D) and may be applicable for prognostic prediction and immunotherapeutic guidance for GC(Li et al., 2018). Hence, we explored whether *MUC16* mutation combined with the IRLPS could yield different prognoses in GC patients. The results showed that the risk groups determined by the IRLPS showed significant survival differences in both the *MUC16* mutation (*MUC16* mut/high vs. *MUC16* mut/low, *p* < 0.0001) and wildtype subgroups (*MUC16* wild/high vs. *MUC16* wild/low, *p* < 0.0001) (Figure 6E), indicating that the predictive ability of the IRLPS was not affected by *MUC16* mutation status. Intriguingly, patients in the *MUC16* mut/high group had a worse OS than patients in the *MUC16* wild/low group (*p* < 0.0001). Moreover, the *MUC16* mut/low group had the best OS rate, and the *MUC16* wild/high group had the worst OS rate (Figure 6E).

### Identification of Potential Compounds Targeting the Immune-Related lncRNA Pairs

**FIGURE 7:**
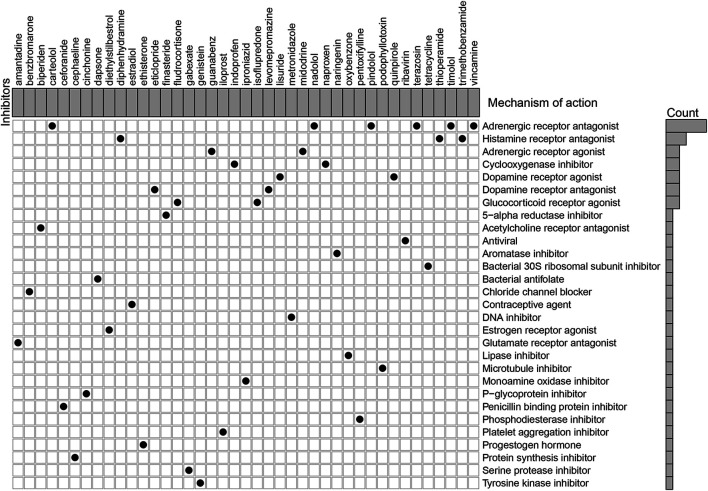
Explorations of candidate drugs targeting the immune-related lncRNA pair signature for the treatment of GC by Connectivity Map analysis.

According to the MoA analysis, there were 29 corresponding molecular pathways targeted by 41 compounds (Figure 7 and Supplementary Table S15). Among them, adrenergic receptor antagonist was the most critical MOA which was shared by six compounds including carteolol, nadolol, pindolol, terazosin, timolol, and vincamine. Then, three compounds (diphenhydramine, thioperamide, and trimethobenzamide) shared the same MoA as histamine receptor antagonist, two compounds (indoprofen and naproxen) shared the same MoA as cyclooxygenase inhibitor, and two compounds (lisuride and quinpirole) shared the same MoA as dopamine receptor agonist. Further studies are warranted to assess the potential therapeutic values of these compounds in GC.

### Development and Evaluation of the Prognostic Nomogram

**FIGURE 8:**
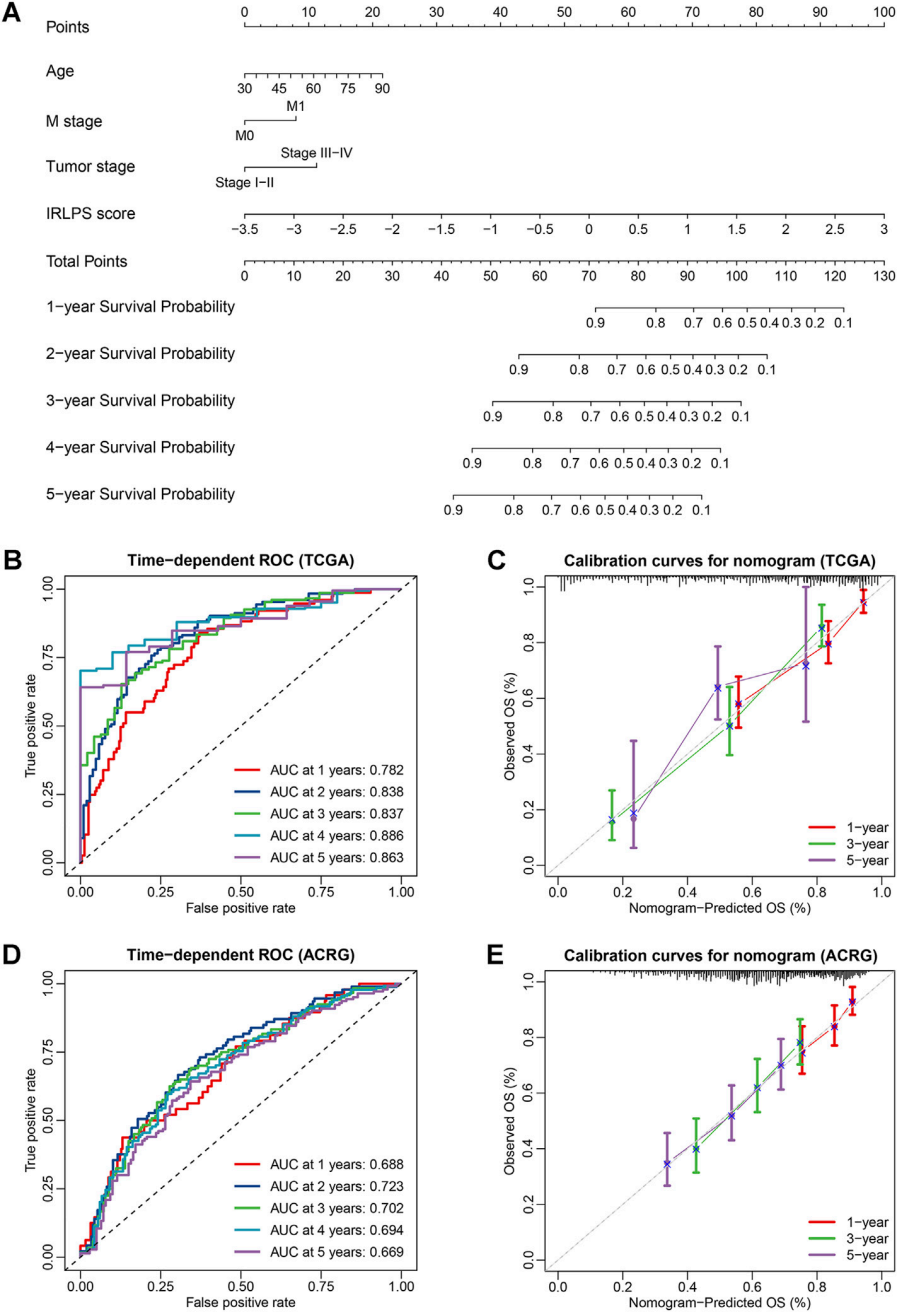
Construction and validation of an individualized nomogram for the overall survival (OS) prediction based on independent prognostic factors. **(A)** A nomogram for the prediction of OS integrating our prognostic signature with independent clinical variables (age, M stage, and tumor stage) was constructed in the TCGA cohort; **(B,D)** Time-dependent receiver operator characteristic (ROC) curves for OS prediction of the nomogram in the TCGA and Asian Cancer Research Group (ACRG) cohorts; **(C,E)** Calibration curves of the nomogram on 1-, 3-, and 5-years survival probability in the TCGA and ACRG cohorts.

Multivariate Cox analysis showed that age, M stage, tumor stage, and IRLPS were independent prognostic factors for the OS in the TCGA cohort. Thus, we developed an individualized nomogram using these variables to further improve the prediction of the OS for GC patients (Figure 8A). The C-index of the nomogram was 0.760 (95% CI: 0.719–0.801) in the TCGA cohort, and similar results were observed when using bootstrapping for the internal validation (C-index: 0.762, 95% CI: 0.722–0.800) (Supplementary Table S16). The nomogram was further validated in the independent ACRG cohort (C-index: 0.653, 95% CI: 0.608–0.698), and it had higher C-index than any other individual included factors such as the tumor stage (Supplementary Table S16). In addition, the ROC curves were also displayed to illustrate the high predictive accuracy of the nomogram (Figures 8B,D). Subsequently, we draw the calibration curves for the OS prediction at 1, 3, and 5 years and revealed the high agreement between the predictions by nomogram and actual observations in both the TCGA and ACRG cohorts (Figures 8C,E). Furthermore, the DCA curves showed that the net benefits of the nomogram were significantly higher than the limit curves (Supplementary Figures S10A, S10B), indicating the good clinical applicability of the nomogram.

## Discussion

GC is a common malignant tumor originating in the digestive tract, of which the prognostic outcome is undesirable despite improved therapeutic strategies (Smyth et al., 2020). Due to the complex tumor heterogeneity and oncogenic mechanisms, developing an individualized prognostic evaluation system for GC that can guide precise clinical treatment remains a challenge. Growing evidence has revealed that tumor progression depends not only on its intrinsic malignant characteristics but also on the TME (Wang et al., 2019). The immune responses in the TME are crucial determinants of tumorigenesis, antitumor immunity, and prognosis (Zeng et al., 2019). As important immune regulators, lncRNAs could impact the prognosis of cancer patients and serve as potential biomarkers for cancer therapies (Zhou et al., 2015; Denaro et al., 2019; Li et al., 2020). Therefore, we focused on the lncRNAs that are correlated with immune modulation to establish the prognostic signature for GC.

Previous studies have published many genetic prognostic models using traditional method that focused on the exact expression levels of coding genes or noncoding RNAs (Wang et al., 2021a; Mao et al., 2021b; Wang et al., 2021c; Xu et al., 2021). However, these signatures may have limited applicability for the evaluation of the prognosis of GC because clinicians have to quantify the gene expression to a standardized unit. Here, we adopted a novel pairing method to transform the lncRNA expression profile to 0-or-1 IRLPs matrix and developed a reasonable prognostic signature named IRLPS in GC (Li et al., 2017). Since the IRLPs were generated entirely based on the relative ranking of their expression in the same patients, there is no need to conduct scaling and normalization to make gene expression levels comparable among patients or datasets. In this study, we enrolled three independent relatively large GC cohorts intending to verify the effectiveness of the method in different populations and measurement platforms. In particular, the lncRNA expression profiles in the GEO microarray datasets were mined by the re-annotation method. Previous studies have shown the feasibility of the profiling of lncRNAs through microarray probe re-annotation (Zhang et al., 2012; Zhou et al., 2015; Wang et al., 2018). Considering the differences of the lncRNA expression profiles extracted from the TCGA and GEO database, we focused on the shared lncRNAs between the two data sources to ensure that the analyzed lncRNAs are general and universal across the GC samples.

A total of 13 IRLPs comprising 26 lncRNAs were selected to construct the prognostic model through the combination of univariate Cox regression, LASSO-Cox regression, and multivariate Cox regression analyses. In the TCGA training cohort, the IRLPS showed good predictive ability for not only the OS but also other survival indicators (DFS, DSS, and PFS) of GC patients, which was effectively validated by bootstrapping technique. Then, subgroup analyses and independent external validations further demonstrated the stability and generalizability of the IRLPS in predicting the OS and DFS of GC patients. Notably, pan-cancer survival analyses also demonstrated the universal applicability of the IRLPS. In the comparisons with the other four published traditional prognostic signatures, we clarified the superiority of the established signature that was based on the paring algorithm. Moreover, the IRLPS was found to be an independent predictive indicator for both the OS and DFS of GC patients. In particular, considering the complementary value between the IRLPS and clinical prognostic characteristics, we further constructed and validated an individualized nomogram which could provide a more accurate prediction of OS for GC and is more convenient for oncologists to use.

Several lncRNAs in the model have been previously confirmed to play important roles in the occurrence and development of GC. For instances, Pan et al. reported that *LIFR-AS1* could contribute to the proliferation and invasion of GC cells *via* miR-29a-3p/COL1A2 axis (Pan et al., 2021). Li et al. (2019) found that *PICART1* functioned as a tumor suppressor by regulating the PI3K/AKT and ERK/MAPK signaling pathways. Zhang et al. (2015) demonstrated that *BANCR* was involved in the growth and apoptosis of GC cells *via* the regulation of NF-κB1, and overexpression of *BANCR* was correlated with unfavorable prognosis of GC patients (Li et al., 2015). In addition, many of the modeling lncRNAs can also contribute to the malignant phenotypes of multiple other cancer types, such as *RBMS3-AS3* in the prostate cancer, *NR2F2-AS1* in the non-small cell lung cancer, and *LINC01554* in the hepatocellular carcinoma (Zhang et al., 2019; Zheng et al., 2019; Jiang et al., 2020). Hence, we believed that the established signature may provide novel biomarkers for the precise treatment of GC.

To elucidate the potential reasons for the survival differences determined by the IRLPS, we first evaluated the associations of the IRLPS with clinical characteristics and found positive correlations between the signature and tumor differentiation, invasion depth, lymph node metastasis, and clinical stage, indicating the essential role of the IRLPS in GC progression. It was known that molecular characteristics were closely related to the prognosis and treatment responses of GC (Cristescu et al., 2015). Here, we observed that GC patients in different molecular subtypes had obviously different risk scores, suggesting that our signature may reflect tumor heterogeneity and participate in the molecular events contributing to the oncogenesis of GC. In particular, the EMT subtype of the ACRG molecular classifier had the highest risk scores and the lowest proportion of predicted responders, which was consistent with previous reports that this subtype predicts poor survival and immunotherapy response (Kim et al., 2018). Next, functional enrichment analyses were performed on the up-regulated genes to further identify the potential mechanisms involved. We observed that the terms correlated to the ECM were markedly enriched in the high-risk group, indicating the active proliferation and migration of tumor cells because the surrounding ECM could influence cell adhesion and polarity and stimulate growth factor signaling (Walker et al., 2018). In addition, several established cancer-related pathways and hallmarks including cAMP and cGMP-PKG signaling pathway, and angiogenesis and EMT related gene sets were enriched in the high-risk group, indicating the internal regulatory mechanisms of the IRLPS in the invasiveness and poor prognosis of GC. It was also worth noting that both the KEGG enrichment analysis and pre-ranked GSEA identified the TGF-β signaling in the high-risk group. Studies have shown that TGF-β could promote the infiltration of T regulatory cells and inhibit the function of effector T cells and natural killer cells, which induces immune suppression within the TME, thus causing tumor immune evasion and poor prognosis in the high-risk group (Batlle and Massagué, 2019). Moreover, we found many immunologic gene sets that were up-regulated in the high-risk group, highlighting the role of IRLPS in regulating the immune system.

Accumulating evidence has recently elucidated the effects of the TME components on defining the immunophenotypes of cancers, which could impact cancer tumorigenesis and prognosis (Wu et al., 2021). Thus, understanding the immune landscape of the TME may help find effective ways to improve patients’ prognoses and therapeutic benefits. To investigate the relationships between the established signature and immune cells, we adopted several acknowledged methods to estimate the TME characteristics. Our results found that tumors with high IRLPS scores exhibited high infiltration levels of monocytes, macrophages, and mast cells, and low infiltration levels of CD4^+^ T cells and follicular T helper cells, which indicated that we could evaluate the immune response of GC tissue according to the IRLPS model. A previous study revealed that tumor-associated macrophages could secrete anti-inflammatory cytokines that induce an immunosuppressive tumor microenvironment by recruiting T regulatory cells, which suppresses the cell-mediated immune response (Mehta et al., 2021). It was also reported that increased tumor-infiltrating mast cells could foster immune suppression and GC progression through TNF-α-PD-L1 pathway (Lv et al., 2019). These may explain the poor prognosis of the high-risk groups. Except for the tumor-infiltrating immune cells, stromal cells also play important roles in shaping the tumor immunophenotypes (Denton et al., 2018). Fortunately, we observed negative correlations between the IRLPS and stromal scores, and the cancer-associated fibroblasts, as the most prominent components within the tumor stroma that could directly contribute to an immunosuppressive environment, are more abundant in the tumors with higher risk scores (Monteran and Erez, 2019). Accordingly, we obtained the expected results of the worse predicted immunotherapy responses in the patients of the high-risk groups by analyzing the TIDE scores and TMB. The TIDE prediction score was associated with the induction of T cell dysfunction in tumors with high cytotoxic T lymphocyte (CTL) levels and the prevention of T cell infiltration in tumors with low CTL levels and thus represents two primary mechanisms of tumor immune escape (Jiang et al., 2018). In this study, higher TIDE and T cell exclusion scores were found in the high-risk groups of all the investigated cohorts, indicating that their lower ICB response rates might be due to the immune evasion through T cell exclusion. In addition, using the somatic mutation data available in the TCGA cohort, we found obviously lower TMB in the high-risk group. It has been reported that tumors with low TMB generates fewer neoantigens, thus avoiding being attacked by CTLs (Wang et al., 2021b). Taken together, these findings suggested the important role of IRLPS in determining TME cell infiltration and predicting response to ICB therapy.

It should be admitted that this study has some limitations. First, although we validated the effectiveness of the signature in two independent GEO cohorts, the overall accuracy of the signature in the validation cohorts was not as good as the TCGA cohort, which may be due to racial differences between different cohorts or sampling bias caused by genomic intratumor heterogeneity (Gyanchandani et al., 2016). Second, the clinical value of the signature in predicting immunotherapy response needs to be assessed in prospective clinical trials. Third, the underlying mechanisms of the identified lncRNAs in the immune regulation of GC have not been fully elucidated, and further research on their functions may contribute to their clinical application as novel therapeutic targets.

In summary, this study proposed an innovative signature for predicting the prognosis of GC patients, which may provide new insights into the role of lncRNAs in tumor immunity and facilitate the more effective development of anti-tumor immunotherapy.

## Data Availability

The datasets presented in this study can be found in online repositories. The names of the repository/repositories and accession number(s) can be found in the article/Supplementary material.
