# Supplementary material for: Comprehensive Analysis of an Individualized Immune-Related lncRNA Pair Signature in Gastric Cancer
Source: Front Cell Dev Biol. 2022 Feb 22;10:805623. doi: 10.3389/fcell.2022.805623 (PMC8902466; doi:10.3389/fcell.2022.805623)
Supplement: Supplementary file 10 [file DataSheet1.docx]

***Supplementary Material***

# Supplementary Figures


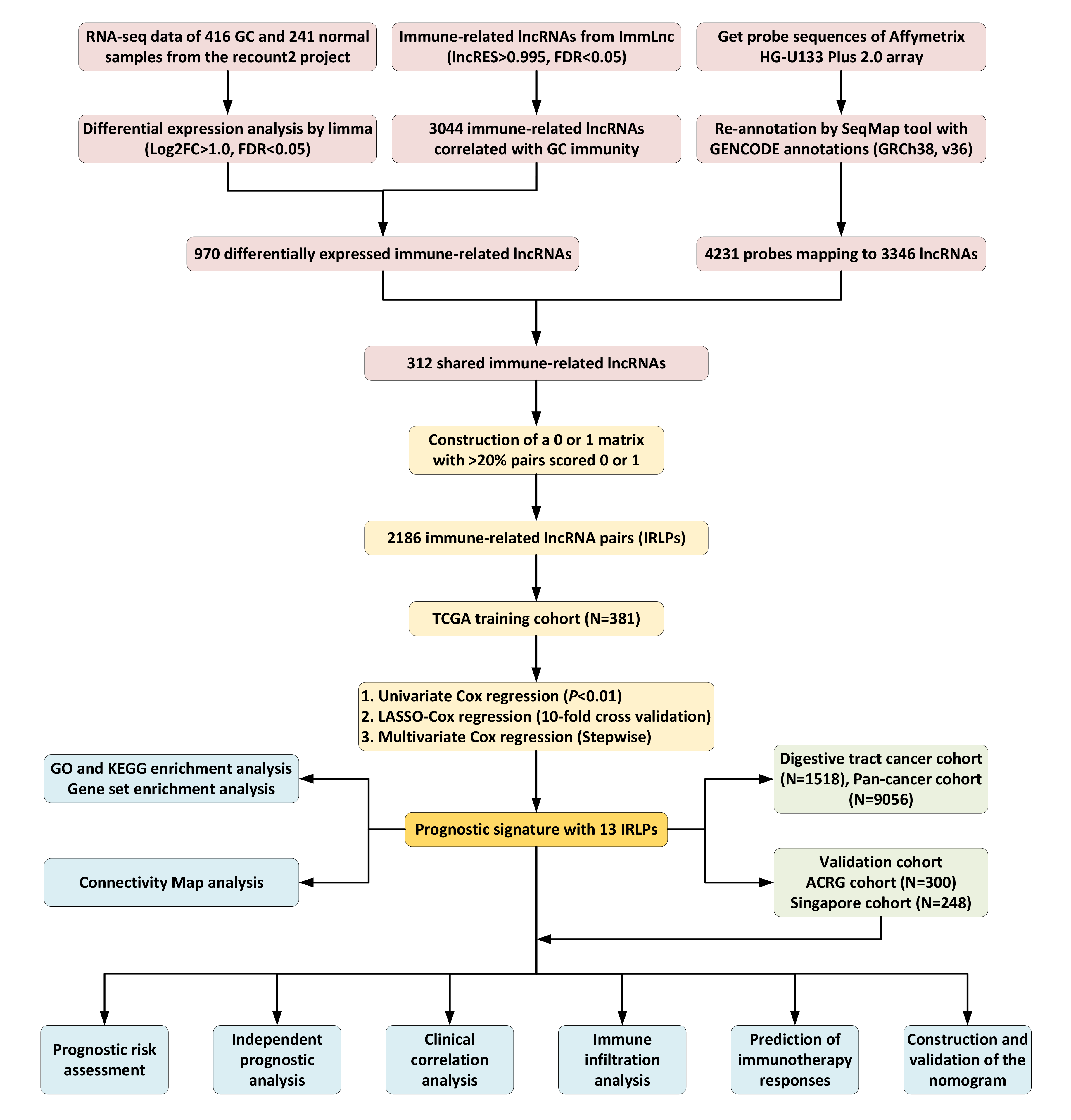


**Supplementary Figure S1. Study flowchart illustrating the process of constructing the prognostic signature to predict prognosis of gastric cancer.**


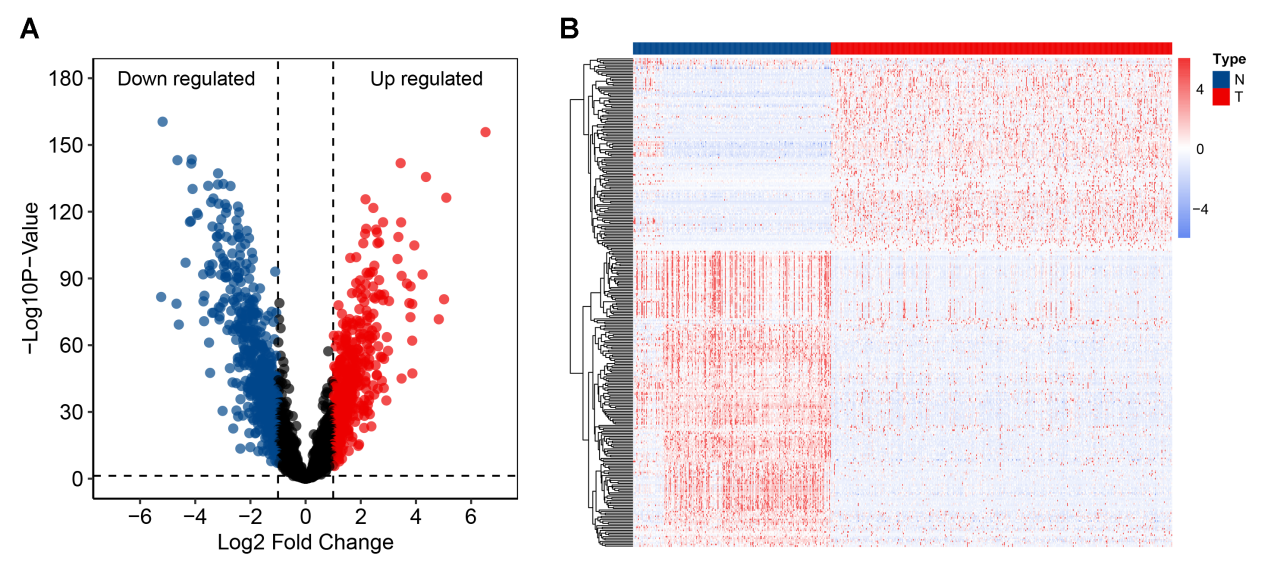


**Supplementary Figure S2. Identification of the differentially expressed immune-related lncRNAs.** **(A)** Volcano plot for the 970 aberrantly expressed immune-related lncRNAs between the 416 GC tissues and 241 (adjacent) normal tissues in The Cancer Genome Atlas (TCGA) and The Genotype-Tissue Expression (GTEx) projects; **(B)** Heatmap for the 312 shared aberrantly expressed immune-related lncRNAs in the TCGA and GTEx samples.


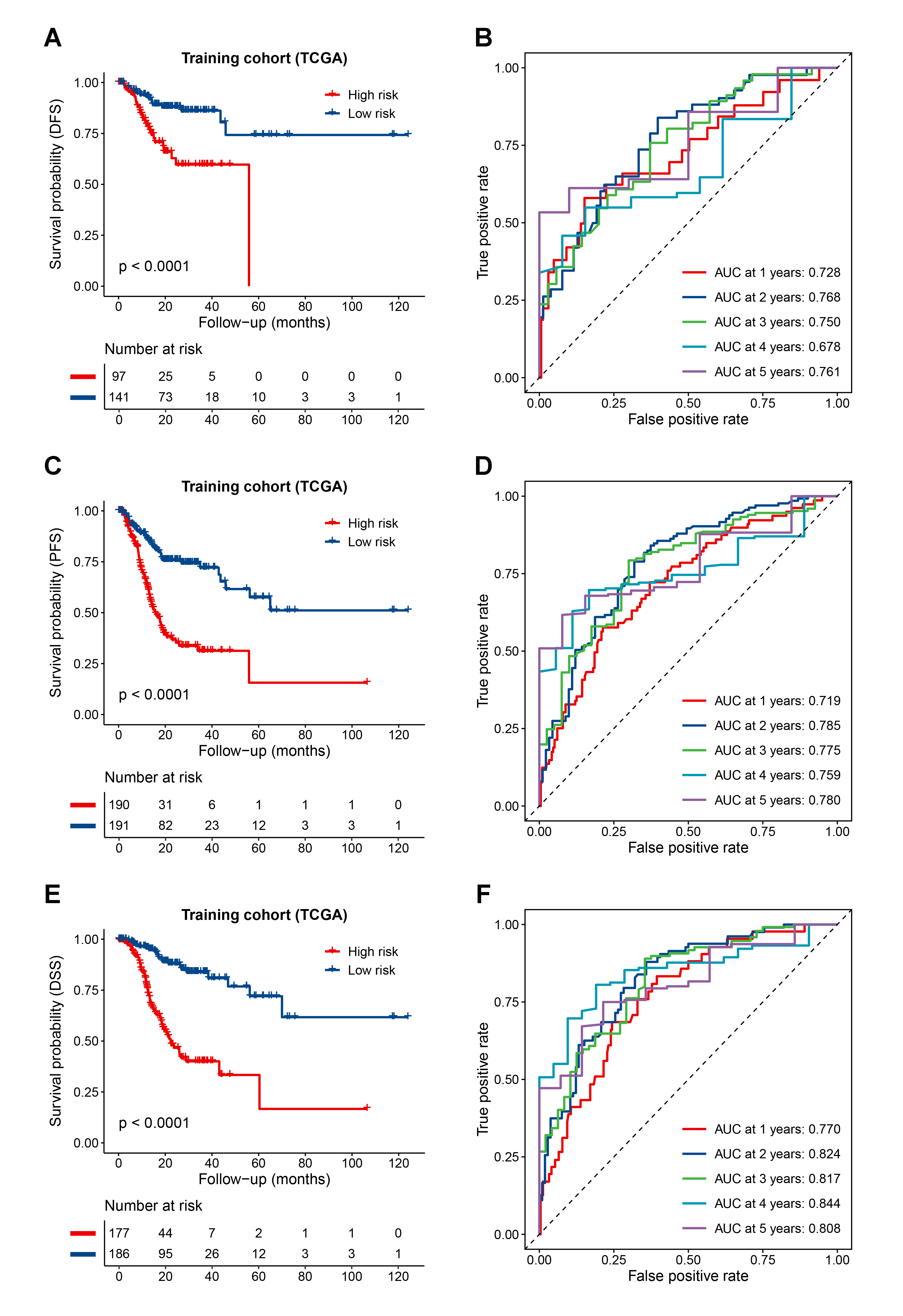


**Supplementary Figure S3. The prognostic assessment of the signature regarding the disease-free survival (DFS), progression-free survival (PFS), and disease-specific survival (DSS) in the TCGA cohort. (A, B)** Kaplan-Meier curves and time-dependent receiver operator characteristic (ROC) curves of the signature for predicting the DFS of GC patients; **(C, D)** Kaplan-Meier curves and time-dependent ROC curves of the signature for predicting the PFS of GC patients; **(E, F)** Kaplan-Meier curves and time-dependent ROC curves of the signature for predicting the DSS of GC patients.


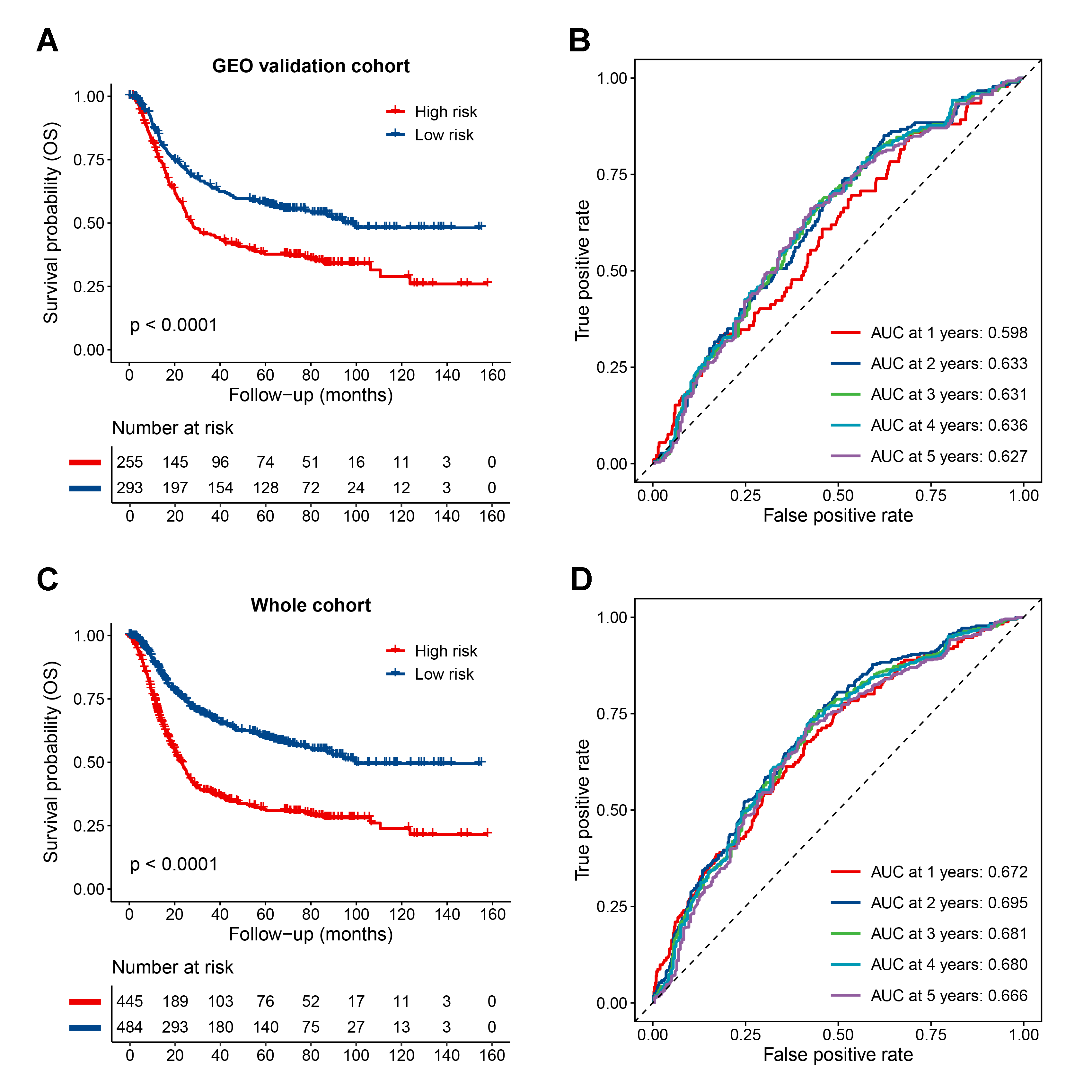


**Supplementary Figure S4. The prognostic assessment of the signature regarding the overall survival (OS) in the GEO validation cohort and the whole cohort. (A, B)** Kaplan-Meier curves and time-dependent receiver operator characteristic (ROC) curves of the signature for predicting the OS of GC patients in the GEO validation cohort (combination of the ACRG and Singapore cohorts); **(C, D)** Kaplan-Meier curves and time-dependent ROC curves of the signature for predicting the OS of GC patients in the whole cohort (combination of the TCGA, ACRG, and Singapore cohorts).


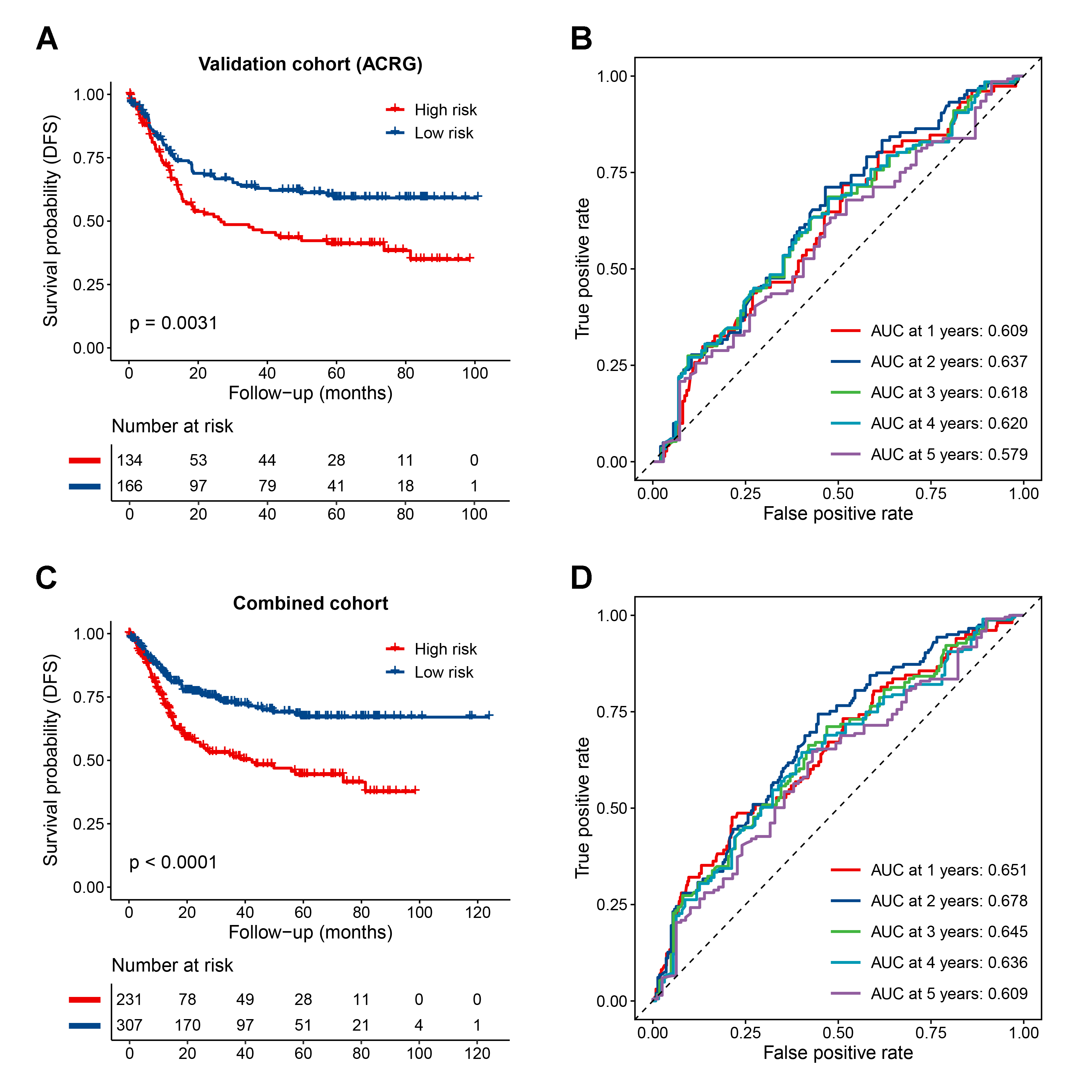


**Supplementary Figure S5. The prognostic assessment of the signature regarding the disease-free survival (DFS) in the ACRG cohort and the combined cohort. (A, B)** Kaplan-Meier curves and time-dependent receiver operator characteristic (ROC) curves of the signature for predicting the DFS of GC patients in the ACRG cohort; **(C, D)** Kaplan-Meier curves and time-dependent ROC curves of the signature for predicting the DFS of GC patients in the combined cohort (combination of the TCGA and ACRG cohorts).


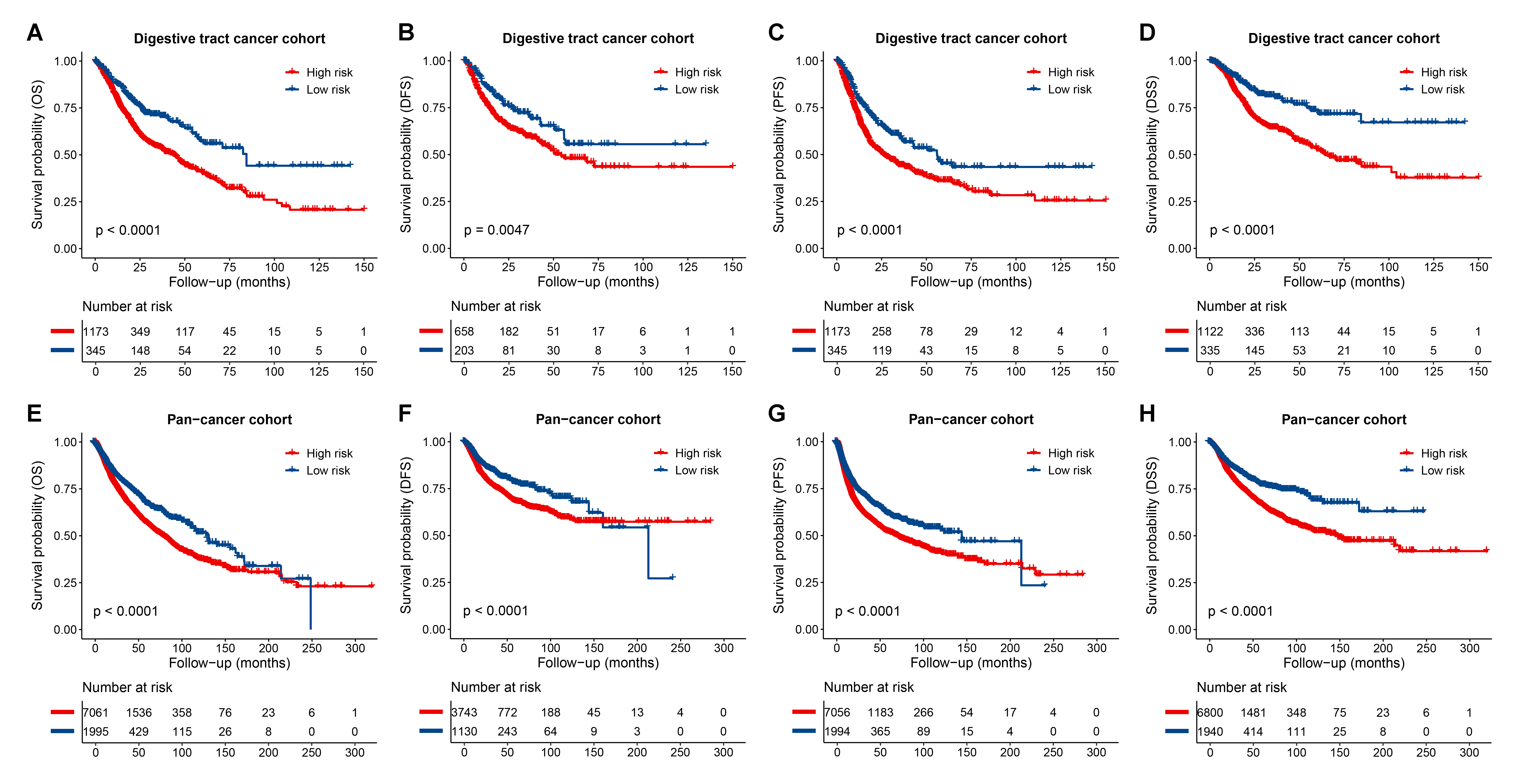


**Supplementary Figure S6. The prognostic assessment of the signature regarding the overall survival (OS), disease-free survival (DFS), progression-free survival (PFS), and disease-specific survival (DSS) using the digestive tract cancer cohort (consolidation of six cancer types with 1518 samples from the TCGA project, including CHOL, COAD, ESCA, LIHC, PAAD, READ, and STAD) and pan-cancer cohort (33 cancer types with 9056 samples).** **(A-D)** Kaplan-Meier curves of the signature for predicting the OS, DFS, PFS, and DSS of cancer patients in the digestive tract cancer cohort; **(E-H)** Kaplan-Meier curves of the signature for predicting the OS, DFS, PFS, and DSS of cancer patients in the pan-cancer cohort. CHOL: cholangiocarcinoma, COAD: colon adenocarcinoma, ESCA: esophageal carcinoma, LIHC: liver hepatocellular carcinoma, PAAD: pancreatic adenocarcinoma, READ: rectum adenocarcinoma, and STAD: stomach adenocarcinoma.


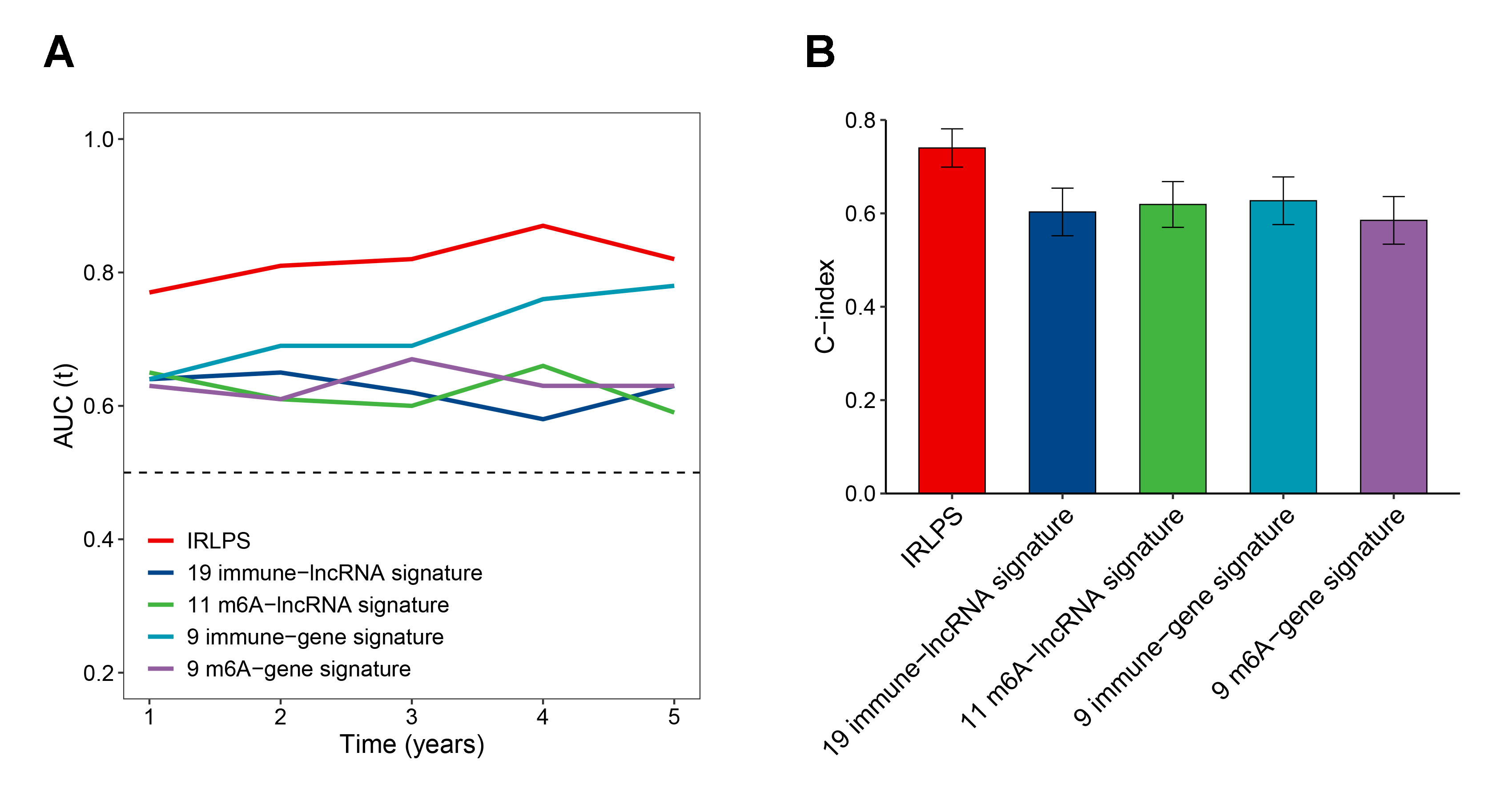


**Supplementary Figure S7. Comparisons of the predictive accuracy of our signature with other four published traditional prognostic signatures in the TCGA cohort.** **(A)** Time-dependent receiver operator characteristic (ROC) curve analyses for different prognostic signatures. The 5-year average AUC values for our immune-related lncRNA pair signature (IRLPS), 19 immune-lncRNA signature, 11 m6A-lncRNA signature, 9 immune-gene signature, 9 m6A-gene signature were 0.817, 0.625, 0.622, 0.714, and 0.635, respectively; **(B)** Concordance index (C-index) analyses for different prognostic signatures. The C-index values and corresponding 95% confidence intervals for the IRLPS, 19-lncRNA signature, 11-lncRNA signature, and 10-lncRNA signature were 0.740 (0.699-0.781), 0.603 (0.552-0.654), 0.619 (0.570-0.668), 0.627 (0.576-0.678), and 0.585 (0.534-0.636), respectively. These results indicate that our IRLPS achieves a higher predictive accuracy than the other traditional signatures.


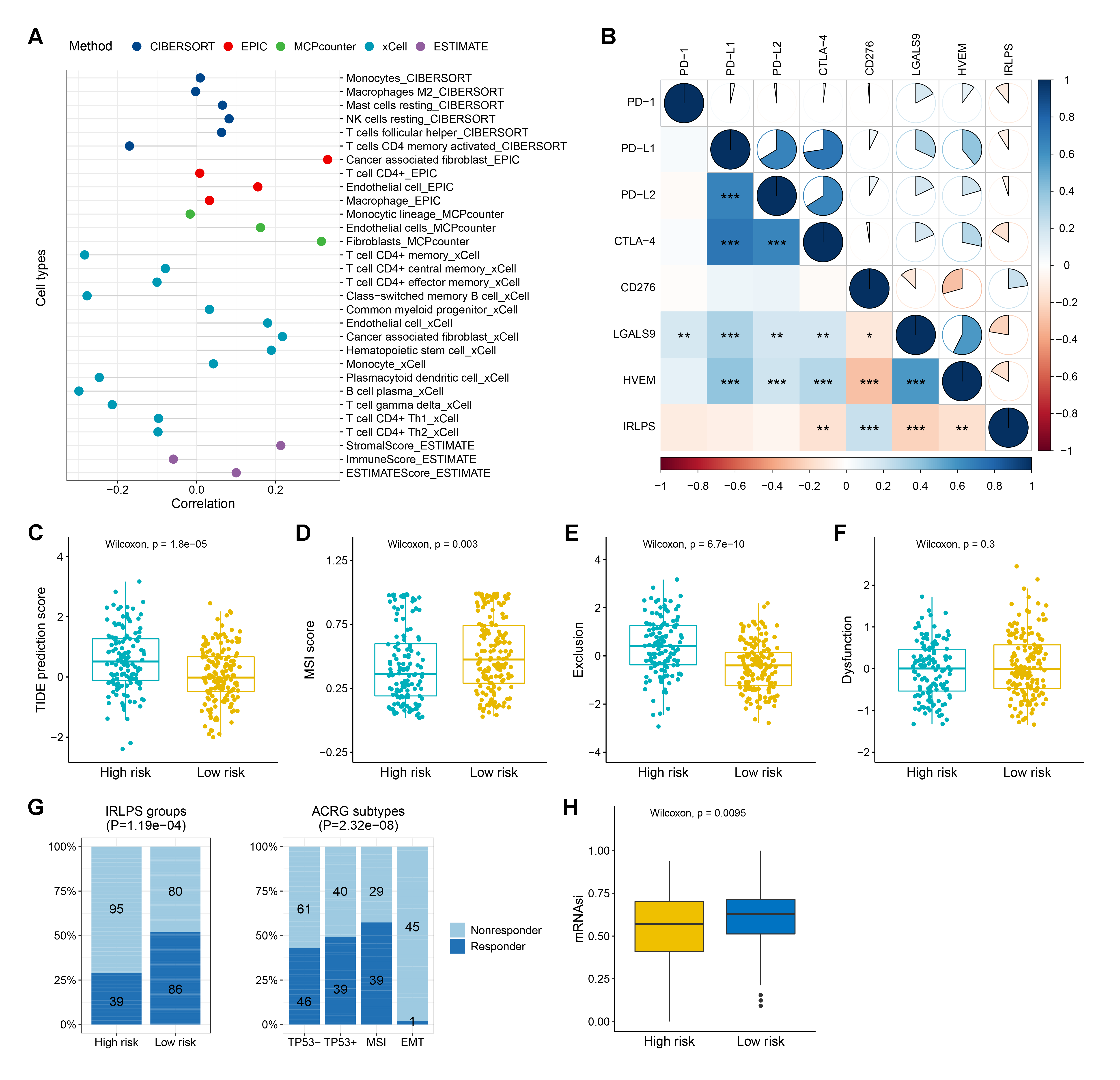


**Supplementary Figure S8. The correlations between the tumor-infiltrating immune cells, immunosuppressed molecules, predicted immunotherapeutic responses, and our prognostic signature in the ACRG cohort. (A)** Lollipop plot displayed the correlations between the signature and tumor-infiltrating immune cells estimated by different algorithms; **(B)** Correlogram showed the correlations between the signature and several crucial immune checkpoint genes, including *PD‐1*, *PD‐L1*, *PD‐L2*, *CTLA‐4*, *CD276*, *LGALS9*, and *HVEM* (correlation coefficients is represented by the area and colored according to the value; **P* < 0.05, ***P* < 0.01, ****P* < 0.001); **(C-F)** Comparisons of the Tumor Immune Dysfunction and Exclusion (TIDE) scores, T cell exclusion scores, dysfunction scores, and microsatellite instability (MSI) scores between the high- and low-risk groups; **(G)** Comparisons of the proportions of predicted responders and non-responders to immunotherapy among different risk groups (left panel) and ACRG subtypes (right panel); **(H)** Boxplot demonstrated the higher stemness index (mRNAsi) in the low-risk group.


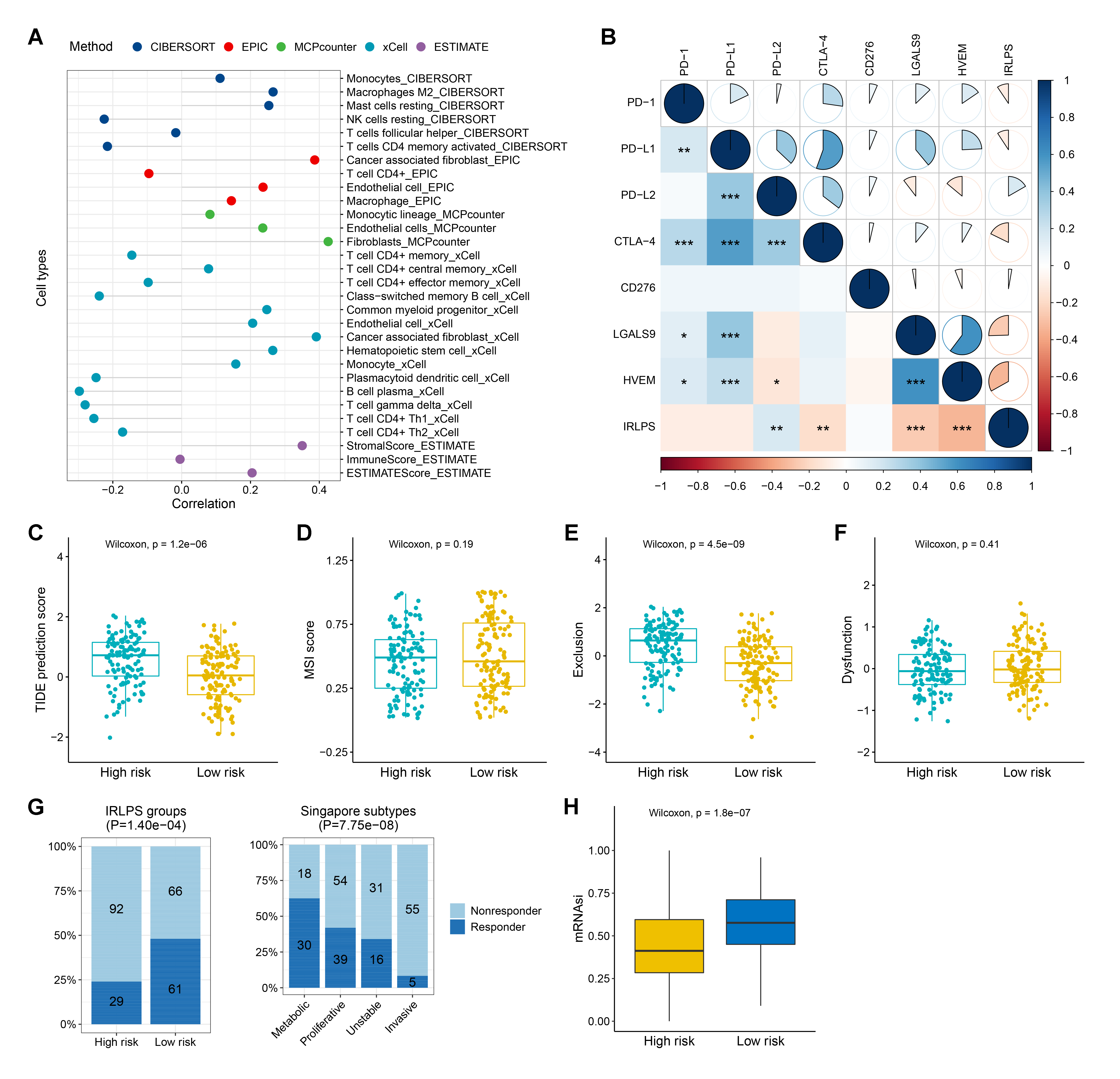


**Supplementary Figure S9. The correlations between the tumor-infiltrating immune cells, immunosuppressed molecules, predicted immunotherapeutic responses, and our prognostic signature in the Singapore cohort.** **(A)** Lollipop plot displayed the correlations between the signature and tumor-infiltrating immune cells estimated by different algorithms; **(B)** Correlogram showed the correlations between the signature and several crucial immune checkpoint genes, including *PD‐1*, *PD‐L1*, *PD‐L2*, *CTLA‐4*, *CD276*, *LGALS9*, and *HVEM* (correlation coefficients is represented by the area and colored according to the value; **P* < 0.05, ***P* < 0.01, ****P* < 0.001); **(C-F)** Comparisons of the Tumor Immune Dysfunction and Exclusion (TIDE) scores, T cell exclusion scores, dysfunction scores, and microsatellite instability (MSI) scores between the high- and low-risk groups; **(G)** Comparisons of the proportions of predicted responders and non-responders to immunotherapy among different risk groups (left panel) and Singapore subtypes (right panel); **(H)** Boxplot demonstrated the higher stemness index (mRNAsi) in the low-risk group.


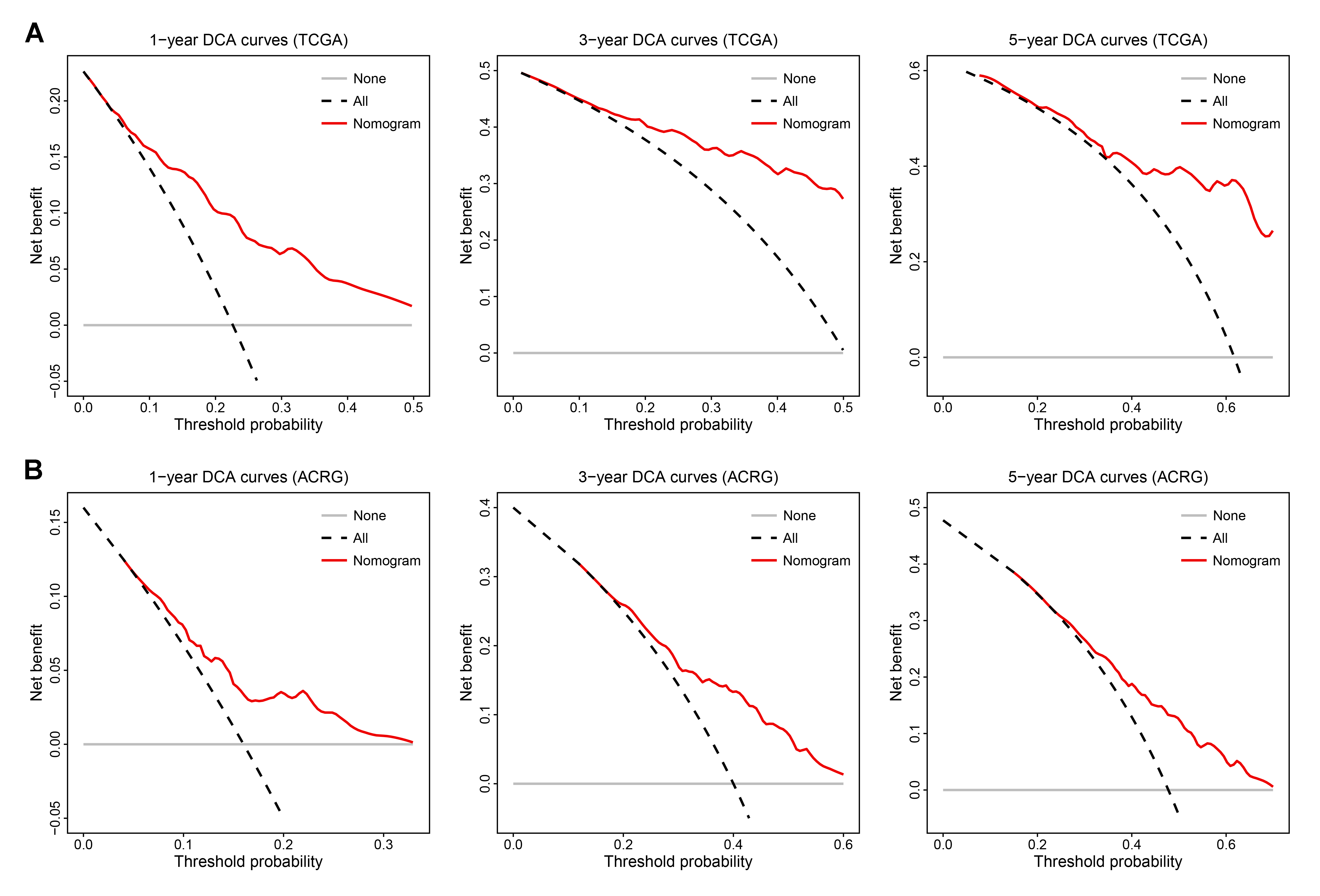


**Supplementary Figure S10. Decision curve analyses (DCA) of the nomogram for 1-, 3-, and 5-year** **overall survival (OS) prediction in the (A)** TCGA cohort and **(B)** Asian Cancer Research Group (ACRG) cohort.

**Supplementary Table S1. Basic demographics and clinical features of GC patients in the TCGA, ACRG and Singapore cohorts.**

| Variables | TCGA cohort (N=381) | ACRG cohort (N=300) | Singapore cohort (N=248) |
| --- | --- | --- | --- |
| Age (mean ± SD) | 65.2±10.7 | 61.9±11.4 | 65.4±12.5 |
| <60 years | 118 (31.0%) | 106 (35.3%) | 66 (26.6%) |
| ≥60 years | 263 (69.0%) | 194 (64.7%) | 182 (73.4%) |
| Gender |  |  |  |
| Male | 249 (65.4%) | 199 (66.3%) | 161 (64.9%) |
| Female | 132 (34.6%) | 101 (33.7%) | 87 (35.1%) |
| Histological grade |  |  |  |
| G1 | 9 (2.4%) | - | - |
| G2 | 133 (34.9%) | - | - |
| G3 | 231 (60.6%) | - | - |
| Gx | 8 (2.1%) | - | - |
| Tumor stage |  |  |  |
| Stage Ⅰ | 53 (13.9%) | 30 (10.0%) | 42 (16.9%) |
| Stage Ⅱ | 123 (32.3%) | 97 (32.3%) | 40 (16.1%) |
| Stage Ⅲ | 168 (44.1%) | 96 (32.0%) | 91 (36.7%) |
| Stage Ⅳ | 37 (9.7%) | 77 (25.7%) | 73 (29.4%) |
| NA | 0 | 0 | 2 (0.8%) |
| T stage |  |  |  |
| T1 | 19 (5.0%) | 0 | - |
| T2 | 80 (21.0%) | 188 (62.7%) | - |
| T3 | 175 (45.9%) | 91 (30.3%) | - |
| T4 | 107 (28.1%) | 21 (7.0%) | - |
| N stage |  |  |  |
| N0 | 115 (30.2%) | 38 (12.7%) | - |
| N1 | 108 (28.3%) | 131 (43.7%) | - |
| N2 | 75 (19.7%) | 80 (26.7%) | - |
| N3 | 78 (20.5%) | 51 (17.0%) | - |
| Nx | 5 (1.3%) | 0 | - |
| M stage |  |  |  |
| M0 | 341 (89.5%) | 273 (91.0%) | - |
| M1 | 24 (6.3%) | 27 (9.0%) | - |
| Mx | 16 (4.2%) | 0 | - |
| Lauren subtype |  |  |  |
| Intestinal | 170 (44.6%) | 146 (48.7%) | 138 (55.6%) |
| Diffuse | 66 (17.3%) | 135 (45.0%) | 86 (34.7%) |
| Mixed | 0 | 19 (6.3%) | 22 (8.9%) |
| NA | 145 (38.1%) ^a^ | 0 | 2 (0.8%) |
| Molecular subtype ^b^ |  |  |  |
|  | EBV (30, 8.7%) | MSS/TP53- (107, 35.7%) | Invasive (60, 24.2%) |
|  | MSI (61, 17.7%) | MSS/TP53+ (79, 26.3%) | Proliferative (93, 37.5%) |
|  | GS (45, 13.0%) | MSI (68, 22.7%) | Metabolic (48, 19.4%) |
|  | CIN (209, 60.6%) | MSS/EMT (46, 15.3%) | Unstable (47, 19.0%) |

GC: gastric cancer, TCGA: The cancer genome atlas, ACRG: Asian cancer research group, SD: standard deviance, EBV: Epstein-Barr virus positive, MSI: microsatellite instability, GS: genomically stable, CIN: chromosomal instability, MSS: microsatellite stable, EMT: epithelial-mesenchymal transition.

^a^ NA or patients of signet ring type or patients not otherwise specified.

^b^ TCGA molecular subtype was obtained using the PanCancerAtlas_subtypes function of the “TCGAbiolinks” R package.

**Supplementary Table S2. R packages used in various analyses.**

| R packages | Function in analyses |
| --- | --- |
| TCGAbiolinks | Data download |
| recount | RNA-seq data preprocessing |
| sva | Removing batch effects |
| limma | Differential expression analysis |
| glmnet | LASSO algorithm |
| survival, survminer | Survival analysis |
| timeROC | Time-dependent ROC analysis |
| ggplot2, ggpubr, ggbeeswarm, corrplot  cowplot, ComplexHeatmap | Visualization |
| CIBERSORT.R, e1071, parallel, preprocessCore  EPIC, MCPcounter, xCell, ESTIMATE | Estimation of immune infiltration |
| gelnet, biomaRt, synapser | One-class logistic regression (OCLR) algorithm |
| clusterProfiler | Go annotation and KEGG enrichment analysis |
| maftools, GenVisR | Mutation data processing |
| rms | Nomogram |
| boot | Internal validation by bootstrap |
| ggDCA | Decision curve analysis |

**Supplementary Table S3. The detailed information about the 312 shared differentially expressed immune-related lncRNAs (submitted as a separate Excel file).**

**Supplementary Table S4. Univariate Cox analysis identified 44 OS-related IRLPs (submitted as a separate Excel file).**

**Supplementary Table S5. The C-index and 95% CIs of the signature in predicting different survival indicators.**

|  | TCGA cohort  (N=381) | Internal validation  (Bootstrap) ^a^ | External validation | |
| --- | --- | --- | --- | --- |
|  |  |  | ACRG cohort  (N=300) | Singapore cohort  (N=248) |
| OS | 0.740  (0.699-0.781) | 0.741  (0.700-0.782) | 0.584  (0.537-0.631) | 0.604  (0.547-0.661) |
| DFS | 0.707  (0.619-0.795) | 0.708  (0.615-0.795) | 0.585  (0.534-0.636) | - |
| DSS | 0.750  (0.699-0.801) | 0.750  (0.699-0.801) | - | - |
| PFS | 0.684  (0.635-0.733) | 0.685  (0.635-0.735) | - | - |

C-index: concordance index, CIs: confidence intervals, TCGA: The cancer genome atlas, ACRG: Asian cancer research group, OS: overall survival, DFS: disease free survival, DSS: disease specific survival, PFS: progression free survival.

^a^ The bootstrap results were displayed as the median and corresponding 2.5th and 97.5th percentile based on 1000 bootstrap replicates.

**Supplementary Table S6. A series of subgroup analyses for the IRLPS in discriminating the OS of patients in the training, validation, and combined cohorts.**

|  | TCGA training cohort (N=381) | | ACRG validation cohort (N=300) | | Singapore validation cohort (N=248) | | GEO validation cohort (N=548) | | Whole cohort (N=929) | |
| --- | --- | --- | --- | --- | --- | --- | --- | --- | --- | --- |
|  | HR (95% CI) | *P* value | HR (95% CI) | *P* value | HR (95% CI) | *P* value | HR (95% CI) | *P* value | HR (95% CI) | *P* value |
| Age |  |  |  |  |  |  |  |  |  |  |
| ≥60 years | 4.094 (2.685-6.241) | 5.76e-11 | 1.407 (0.956-2.072) | 0.083 | 1.806 (1.173-2.779) | 0.007 | 1.591 (1.194-2.119) | 0.002 | 2.155 (1.709-2.717) | 8.58e-11 |
| <60 years | 5.739 (2.597-12.680) | 1.57e-05 | 2.271 (1.282-4.021) | 0.005 | 1.524 (0.771-3.011) | 0.226 | 2.038 (1.316-3.157) | 0.001 | 2.732 (1.874-3.982) | 1.71e-07 |
| Gender |  |  |  |  |  |  |  |  |  |  |
| Male | 4.178 (2.710-6.442) | 9.51e-11 | 1.639 (1.102-2.436) | 0.015 | 2.202 (1.398-3.470) | 6.64e-04 | 1.869 (1.387-2.519) | 3.94e-05 | 2.391 (1.876-3.048) | 1.91e-12 |
| Female | 5.256 (2.534-10.900) | 8.28e-06 | 1.775 (1.028-3.065) | 0.040 | 1.124 (0.611-2.067) | 0.706 | 1.450 (0.966-2.176) | 0.073 | 2.118 (1.509-2.971) | 1.41e-05 |
| Tumor stage |  |  |  |  |  |  |  |  |  |  |
| Stage Ⅰ-Ⅱ | 4.065 ( 2.169-7.618) | 1.21e-05 | 1.648 (0.857-3.172) | 0.135 | 1.603 (0.650-3.952) | 0.306 | 1.610 (0.948-2.734) | 0.078 | 2.368 (1.593-3.519) | 2.03e-05 |
| Stage Ⅲ-Ⅳ | 4.584 (2.847- 7.380) | 3.68e-10 | 1.550 (1.074-2.236) | 0.019 | 1.520 (1.017-2.272) | 0.041 | 1.576 (1.203-2.065) | 9.70e-04 | 2.115 (1.682-2.659) | 1.46e-10 |
| Histological grade |  |  |  |  |  |  |  |  |  |  |
| G1-2 | 4.866 (2.493-9.498) | 3.53e-06 | - | - | - | - | - | - | 4.866 (2.493-9.498) | 3.53e-06 |
| G3 | 4.636 (2.872-7.485) | 3.43e-10 | - | - | - | - | - | - | 4.636 (2.872-7.485) | 3.43e-10 |
| T stage |  |  |  |  |  |  |  |  |  |  |
| T1-2 | 2.591 (1.208-5.557) | 0.015 | 1.996 (1.265-3.147) | 0.003 | - | - | 1.996 (1.265-3.147) | 0.003 | 2.112 (1.429-3.120) | 1.74e-04 |
| T3-4 | 5.508 (3.524-8.611) | 7.13e-14 | 1.190 (0.760-1.865) | 0.447 | - | - | 1.190 (0.760-1.865) | 0.447 | 2.707 (2.009-3.648) | 6.11e-11 |
| N stage |  |  |  |  |  |  |  |  |  |  |
| N0 | 5.744 (2.571-12.840) | 2.03e-05 | 4.433 (1.106-17.770) | 0.036 | - | - | 4.433 (1.106-17.770) | 0.036 | 4.549 (2.317-8.932) | 1.08e-05 |
| N1-3 | 3.885 (2.536-5.954) | 4.58e-10 | 1.492 (1.074-2.072) | 0.017 | - | - | 1.492 (1.074-2.072) | 0.017 | 2.194 (1.706-2.821) | 9.23e-10 |
| M stage |  |  |  |  |  |  |  |  |  |  |
| M0 | 4.272 (2.880-6.337) | 5.26e-13 | 1.674 (1.180-2.374) | 0.004 | - | - | 1.674 (1.180-2.374) | 0.004 | 2.521 (1.953-3.253) | 1.18e-12 |
| M1 | 4.619 (1.251-17.050) | 0.022 | 2.430 (1.074-5.500) | 0.033 | - | - | 2.430 (1.074-5.500) | 0.033 | 3.250 (1.641-6.435) | 7.23e-04 |
| Lauren subtype |  |  |  |  |  |  |  |  |  |  |
| Intestinal | - | - | 2.101 (1.256-3.513) | 0.005 | 2.286 (1.375-3.799) | 0.001 | 2.233 (1.557-3.202) | 1.24e-05 | 2.233 (1.557-3.202) | 1.24e-05 |
| Diffuse | - | - | 1.306 (0.839-2.032) | 0.237 | 1.236 (0.691-2.211) | 0.476 | 1.301 (0.915-1.848) | 0.143 | 1.301 (0.915-1.848) | 0.143 |
| Mixed | - | - | 1.772 (0.577-5.440) | 0.318 | 1.121 (0.298-4.217) | 0.866 | 1.475 (0.630-3.457) | 0.371 | 1.475 (0.630-3.457) | 0.371 |

IRLPS: immune-related lncRNA pair signature; OS: overall survival; TCGA: The cancer genome atlas, ACRG: Asian cancer research group; HR: hazard ratio; CI: confidence interval.

Bonferroni correction with *P*<0.025 was considered to be statistically significant.

**Supplementary Table S7. A series of subgroup analyses for the IRLPS in discriminating the DFS of patients in the training, validation, and combined cohorts.**

|  | TCGA training cohort (N=238) | | ACRG validation cohort (N=300) | | Combined cohort (N=538) | |
| --- | --- | --- | --- | --- | --- | --- |
|  | HR (95% CI) | *P* value | HR (95% CI) | *P* value | HR (95% CI) | *P* value |
| Age |  |  |  |  |  |  |
| ≥60 years | 2.239 (1.094-4.584) | 0.027 | 1.356 (0.869-2.116) | 0.180 | 1.609 (1.104-2.346) | 0.013 |
| <60 years | 7.959 (2.119-29.900) | 0.002 | 2.497 (1.382-4.513) | 0.002 | 3.196 (1.875-5.447) | 1.94e-05 |
| Gender |  |  |  |  |  |  |
| Male | 3.021 (1.501-6.083) | 0.002 | 1.781 (1.148-2.763) | 0.010 | 2.151 (1.485-3.116) | 5.14e-05 |
| Female | 4.696 (1.213-18.180) | 0.025 | 1.584 (0.867-2.893) | 0.135 | 1.806 (1.058-3.085) | 0.030 |
| Tumor stage |  |  |  |  |  |  |
| Stage Ⅰ-Ⅱ | 2.881 (1.271-6.533) | 0.011 | 1.168 (0.524-2.601) | 0.705 | 1.774 (1.013-3.108) | 0.045 |
| Stage Ⅲ-Ⅳ | 3.639 (1.427-9.283) | 0.007 | 1.704 (1.146-2.534) | 0.008 | 2.040 (1.417-2.937) | 1.26e-04 |
| Histological grade |  |  |  |  |  |  |
| G1-2 | 2.957 (0.932-9.382) | 0.066 | - | - | 2.957 (0.932-9.382) | 0.066 |
| G3 | 3.088 (1.499-6.361) | 0.002 | - | - | 3.088 (1.499-6.361) | 0.002 |
| T stage |  |  |  |  |  |  |
| T1-2 | 1.174 (0.340-4.055) | 0.800 | 2.000 (1.187-3.372) | 0.009 | 1.851 (1.149-2.982) | 0.011 |
| T3-4 | 4.912 (2.258-10.680) | 5.97e-05 | 1.168 (0.726-1.882) | 0.522 | 2.065 (1.389-3.069) | 3.40e-04 |
| N stage |  |  |  |  |  |  |
| N0 | 4.963 (1.819-13.540) | 0.002 | 5.698 (1.103-29.420) | 0.038 | 5.090 (2.174-11.920) | 1.78e-04 |
| N1-3 | 2.468 (1.131-5.386) | 0.023 | 1.511 (1.052-2.170) | 0.025 | 1.665 (1.200-2.309) | 0.002 |
| M stage |  |  |  |  |  |  |
| M0 | 3.338 (1.779-6.264) | 1.74e-04 | 1.728 (1.177-2.537) | 0.005 | 2.110 (1.524-2.921) | 6.93e-06 |
| M1 | - | - | 1.785 (0.715-4.458) | 0.215 | 1.785 (0.715-4.458) | 0.215 |
| Lauren subtype |  |  |  |  |  |  |
| Intestinal | - | - | 2.590 (1.456-4.609) | 0.001 | 2.590 (1.456-4.609) | 0.001 |
| Diffuse | - | - | 1.276 (0.790-2.062) | 0.319 | 1.276 (0.790-2.062) | 0.319 |
| Mixed | - | - | 1.014 (0.265-3.885) | 0.984 | 1.014 (0.265-3.885) | 0.984 |

IRLPS: immune-related lncRNA pair signature; DFS: disease free survival; TCGA: The cancer genome atlas, ACRG: Asian cancer research group; HR: hazard ratio; CI: confidence interval.

Bonferroni correction with *P*<0.025 was considered to be statistically significant.

**Supplementary Table S8. Univariate and multivariate Cox regression analysis of clinicopathologic factors and IRLPS for predicting DFS in the training and validation cohorts.**

| For DFS variables | Univariate analysis | |  | Multivariate analysis | |
| --- | --- | --- | --- | --- | --- |
|  | HR (95% CI) | *P* value |  | HR (95% CI) | *P* value |
| TCGA training cohort (N=238) ^a^ |  |  |  |  |  |
| Age (≥60 vs. <60 years) | 1.244 (0.648-2.388) | 0.511 |  | - | - |
| Gender (Male vs. Female) | 1.997 (1.007-3.959) | 0.048 |  | 2.141 (1.078-4.251) | 0.030 |
| Histological grade (G3 vs. G1-2) | 1.790 (0.921-3.481) | 0.086 |  | - | - |
| T stage (T3-4 vs. T1-2) | 1.247 (0.626-2.484) | 0.529 |  | - | - |
| N stage (N1-3 vs. N0) | 1.035 (0.564-1.898) | 0.913 |  | - | - |
| Tumor stage (Stage Ⅲ-Ⅳ vs. Stage Ⅰ-Ⅱ) | 1.019 (0.562-1.847) | 0.951 |  | - | - |
| IRLPS (high‐ vs. low‐risk) | 3.222 (1.737-5.976) | 2.05e-04 |  | 3.342 (1.804-6.192) | 1.26e-04 |
| ACRG validation cohort (N=300) |  |  |  |  |  |
| Age (≥60 vs. <60 years) | 1.096 (0.763-1.574) | 0.621 |  | - | - |
| Gender (Male vs. Female) | 0.959 (0.663-1.388) | 0.825 |  | - | - |
| T stage (T3-4 vs. T1-2) | 2.562 (1.799-3.647) | 1.80e-07 |  | 1.321 (0.852-2.048) | 0.214 |
| N stage (N1-3 vs. N0) | 3.033 (1.414-6.509) | 0.004 |  | 1.813 (0.810-4.059) | 0.148 |
| M stage (M1 vs. M0) | 3.844 (2.365-6.246) | 5.46e-08 |  | 2.755 (1.652-4.595) | 1.03e-04 |
| Lauren subtype (Diffused vs. Intestinal) | 1.559 (1.078-2.254) | 0.018 |  | 1.032 (0.697-1.528) | 0.876 |
| Lauren subtype (Mixed vs. Intestinal) | 1.898 (0.931-3.869) | 0.078 |  | 1.455 (0.709-2.985) | 0.307 |
| Tumor stage (Stage Ⅲ-Ⅳ vs. Stage Ⅰ-Ⅱ) | 4.186 (2.696-6.500) | 1.79e-10 |  | 2.668 (1.535-4.635) | 5.00e-04 |
| IRLPS (high‐ vs. low‐risk) | 1.691 (1.189-2.404) | 0.003 |  | 1.600 (1.118-2.290) | 0.010 |

IRLPS: immune-related lncRNA pair signature; DFS: disease free survival; HR: hazard ratio; CI: confidence interval; TCGA: The cancer genome atlas, ACRG: Asian cancer research group.

^a^ Patients with Gx (histological grade cannot be assessed) or Nx (lymph nodes cannot be assessed) or Mx (distant metastasis cannot be assessed) were included in the analysis but were not displayed in this table (coefficients were infinite), and the TCGA DFS dataset has no patients of the M1 stage.

**Supplementary Table S9. 165 differentially expressed genes between the high- and low-risk groups of the TCGA cohort (submitted as a separate Excel file).**

**Supplementary Table S10. GO annotations of the significantly up-regulated genes in the high-risk group of the TCGA cohort (submitted as a separate Excel file).**

**Supplementary Table S11. KEGG enrichment of the significantly up-regulated genes in the high-risk group of the TCGA cohort (submitted as a separate Excel file).**

**Supplementary Table S12. GSEA analysis for cancer hallmarks positively correlated with the high-risk group of the TCGA cohort (submitted as a separate Excel file).**

**Supplementary Table S13. GSEA analysis for immunologic characteristics positively correlated with the high-risk group of the TCGA cohort (submitted as a separate Excel file).**

**Supplementary Table S14. The detailed results of the correlation analysis between the tumor-infiltrating immune cells and the prognostic signature (submitted as a separate Excel file).**

**Supplementary Table S15. Detailed information of the candidate drugs identified by the Connectivity Map (CMap) database (submitted as a separate Excel file).**

**Supplementary Table S16. The C-index and 95% CIs of the nomogram and single included factors in predicting the OS.**

|  | TCGA cohort ^a^  (N=365) | Internal validation  (Bootstrap) ^b^ | External validation  (ACRG cohort, N=300) |
| --- | --- | --- | --- |
| Nomogram | 0.760 (0.719-0.801) | 0.762 (0.722-0.800) | 0.653 (0.608-0.698) |
| IRLPS | 0.733 (0.692-0.774) | 0.735 (0.692-0.774) | 0.584 (0.537-0.631) |
| Tumor stage | 0.593 (0.552-0.634) | 0.593 (0.550-0.635) | 0.641 (0.606-0.676) |
| M stage | 0.538 (0.509-0.567) | 0.538 (0.508-0.567) | 0.562 (0.537-0.587) |
| Age | 0.549 (0.496-0.602) | 0.551 (0.501-0.603) | 0.549 (0.500-0.598) |

C-index: concordance index, CIs: confidence intervals, OS: overall survival, TCGA: The cancer genome atlas, ACRG: Asian cancer research group, IRLPS: immune-related lncRNA pair signature.

^a^ A total of 365 GC patients were included to construct the nomogram in the TCGA cohort since we have excluded the patients with Mx (distant metastasis cannot be assessed).

^b^ The bootstrap results were displayed as the median and corresponding 2.5th and 97.5th percentile based on 1000 bootstrap replicates.
